# Supplementary material for: Community-based intervention for monitoring of salt intake in hypertensive patients: A cluster randomized controlled trial
Source: PLoS One. 2024 Nov 22;19(11):e0311908. doi: 10.1371/journal.pone.0311908 (PMC11584128; doi:10.1371/journal.pone.0311908)
Supplement: S1 Questionnaire — In Thai. (PDF) [file pone.0311908.s002.pdf]

แบบสอบถาม

เรื่อง การทดลองควบคุมแบบสุ่มแบบคลัสเตอร์ต่อการบริโภคเกลือโซเดียมและความดันโลหิตในผู้ป่วยโรคความดันโลหิตสูงในชุมชน

คำชี้แจงก่อนตอบแบบสอบถาม

แบบสอบถามนี้ใช้ประกอบการพัฒนารูปแบบชุมชนลดเค็ม ลดโรคไม่ติดต่อเรื้อรัง เขตภาคเหนือ จำนวน 46 ข้อ ประกอบด้วย 5 ส่วน ดังนี้

ส่วนที่ 1 แบบสอบถามเกี่ยวกับข้อมูลทั่วไป จำนวน 5 ข้อ

ส่วนที่ 2 ผลการตรวจร่างกาย จำนวน 4 ข้อ

ส่วนที่ 3 แบบประเมินความรู้ และการรับรู้ในความรุนแรงเกี่ยวกับเกี่ยวกับโรคไม่ติดต่อเรื้อรัง จำนวน 10 ข้อ

ส่วนที่ 4 แบบสอบถามทัศนคติเกี่ยวกับพฤติกรรมที่เกี่ยวข้องกับ โรคเบาหวาน โรคความดันโลหิตสูง และโรคไตวาย จำนวน 13 ข้อ

ส่วนที่ 5 แบบสอบถามพฤติกรรมการป้องกันโรค จำนวน 14 ข้อ

ในการกรอกแบบสอบถามขอความกรุณาให้ท่านตอบให้ครบทุกข้อคำถามข้อมูลที่ได้จากท่านทั้งหมด ผู้วิจัยจะเก็บไว้เป็นความลับ ข้อมูลทั้งหมดที่ได้จะนำมาสรุปและนำเสนอผลการดำเนินงานในเชิงวิชาการเท่านั้น

ขอขอบคุณ มา ณ โอกาสนี้

การทดลองควบคุมแบบสุ่มแบบคลัสเตอร์ต่อการบริโภคเกลือโซเดียมและความดันโลหิตในผู้ป่วยโรคความดันโลหิตสูงในชุมชน

ส่วนที่ 1 ข้อมูลทั่วไป

คำชี้แจง กรุณาทำเครื่องหมาย ✓ ลงใน ☐ หรือเติมข้อความในช่องว่างที่ตรงตามความเป็นจริงในตัวท่าน

1. ระดับการศึกษาสูงสุด

☐ 1. ไม่ได้เรียนหนังสืออ่านเขียนไม่ได้ ☐ 2. ไม่ได้เรียน/ไม่จบประถมศึกษาแต่อ่านออกเขียนได้

☐ 3. ประถมศึกษา ☐ 4. มัธยมศึกษาตอนต้น

☐ 5. มัธยมศึกษาตอนปลาย/ปวช. ☐ 6. อนุปริญญา/ปวส.

☐ 7. ปริญญาตรีขึ้นไป

2. อาชีพหลักของท่าน (เลือกตอบ 1 ข้อ เท่านั้น)

☐ 1. แม่บ้าน ☐ 2. เกษตรกร (ทำไร่ ทำสวน ทำนา) ☐ 3. ค้าขาย

☐ 4. รับราชการ ☐ 5. รับจ้าง ☐ 6. อื่นๆ ระบุ..................

3. ท่านเป็นโรคความดันโลหิตสูงและโรคอื่นๆ (เลือกตอบได้มากกว่า 1 ข้อ)

☐ 1. เบาหวาน ☐ 2. ไขมันในเลือดสูง

☐ 3. หัวใจ ☐ 4. โรคไตเรื้อรัง (ยกเว้นระยะสุดท้าย)

☐ 5. หลอดเลือดสมอง ☐ 6. ปอดอุดกั้นเรื้อรัง

☐ 7. อื่นๆระบุ.............. ☐ 8. ไม่มี

4. เดือนที่ผ่านมาท่านดื่มแอลกอฮอล์

☐1.ดื่ม ☐ 2. ไม่ดื่ม

☐ 3. เคยดื่มแต่เลิกแล้ว .... ปี

5. ท่านสูบบุหรี่

☐ 1. สูบ ☐ 2. ไม่สูบ

☐ 3. เคยสูบแต่เลิกแล้ว .... ปี

ส่วนที่ 2 ผลการตรวจร่างกาย

คำชี้แจง กรุณาทำเครื่องหมาย ✓ ลงใน ☐ หรือเติมข้อความในช่องว่างที่ตรงตามความเป็นจริงในตัวท่าน ผลการตรวจร่างกาย/ประเมินพฤติกรรมเสี่ยง ณ วันที่ไปประเมิน

1. น้ำหนัก..กิโลกรัม
2. ส่วนสูง..เซนติเมตร
3. เส้นรอบเอว.....................................เซนติเมตร

4. ความดันโลหิต

ครั้งที่ 1................../.................มิลลิเมตรปรอท

ครั้งที่ 2................../.................มิลลิเมตรปรอท

ครั้งที่ 3................../.................มิลลิเมตรปรอท

ส่วนที่ 3 แบบประเมินความรู้ และการรับรู้ในความรุนแรงเกี่ยวกับเกี่ยวกับโรคไม่ติดต่อเรื้อรัง

คำชี้แจง โปรดทำเครื่องหมาย ✓ ลงในช่องว่าง ที่ตรงกับความเป็นจริงของท่านมากที่สุด ในแต่ละข้อคำถาม และทำให้ครบทุกข้อ

ข้อความ	ใช่	ไม่ใช่	~~ไม่แน่ใจ~~
1. เกลือ มีโซเดียมเป็นส่วนประกอบ ร่างกายต้องการโซเดียมเพียง 2,000 มิลลิกรัมต่อวัน หรือเท่ากับเกลือ 1 ช้อนชาเท่านั้น			
2. คนไทยบริโภคโซเดียมสูงเกินกว่าที่ร่างกายต้องการถึง 2 เท่าต่อวัน ซึ่งเป็นสาเหตุสำคัญของการเกิดโรคไม่ติดต่อเรื้อรังโดยเฉพาะ โรคความดันโลหิตสูง โรคไต โรคหัวใจและหลอดเลือดสมอง			
3. แหล่งอาหารที่มีโซเดียมสูง ได้แก่ เครื่องปรุงรส (น้ำปลา น้ำซีอิ้ว เกลือ ซอสหอยนางรม ผงชูรส รสดีซุปก้อน น้ำจิ้มสุกี้) และ อาหารแปรรูปหรือถนอมอาหาร (ไส้กรอก ลูกชิ้น อาหารกระป๋อง ผักดอง ผลไม้ดอง กะปิ ปลาร้า เต้าเจี้ยว น้ำบูดู)			
4. อาหารตามธรรมชาติล้วนมีโซเดียมอยู่ทั้งสิ้น ไม่ว่าจะเป็น ข้าวสวย ผลไม้ เนื้อสัตว์ เป็นต้น			
5. ฉลากอาหารที่ติดข้างภาชนะบรรจุ มีข้อมูลส่วนประกอบของอาหาร, ปริมาณสารอาหาร ที่สำคัญ คือ ปริมาณโซเดียม น้ำตาล ไขมัน ฯลฯ ซึ่งเราควรศึกษาก่อนซื้อ			
6. คนที่อ้วนลงพุงแต่ไม่มีกรรมพันธุ์เป็นโรคความดันโลหิตสูงและเบาหวาน จะไม่เสี่ยงต่อการเกิดโรคความดันโลหิตสูงและโรคเบาหวาน			
7. โรคความดันโลหิตสูง และโรคเบาหวาน เป็นสาเหตุสำคัญของโรคไตวาย			
8. การกินเค็มทำให้อัตราการกรองของเสียผ่านไตมากขึ้น ทำให้ไตแข็งแรงมากขึ้น			
9. การเติมความหวานลงในอาหารช่วยทำให้กลบรสชาติความเค็ม ทำให้ช่วยลดปริมาณโซเดียม ในอาหารลงได้			
10. การลดน้ำหนัก การกินผักอย่างเพียงพอ และออกกำลังกายอย่างสม่ำเสมอไม่ช่วยทำให้ระดับความดันโลหิตลดลง			

ส่วนที่ 4 แบบสอบถามทัศนคติเกี่ยวกับพฤติกรรมที่เกี่ยวข้องกับ โรคเบาหวาน โรคความดันโลหิตสูง และโรคไตวาย

คำชี้แจง โปรดทำเครื่องหมาย ✓ ลงในช่องว่าง ที่ตรงกับความเป็นจริงของท่านมากที่สุด ในแต่ละข้อคำถาม และทำให้ครบทุกข้อ

การรับรู้ประโยชน์ และการรับรู้อุปสรรคของพฤติกรรมการป้องกันโรค	ระดับ				
	เห็นด้วยมากที่สุด	เห็นด้วยมาก	เห็นด้วยปานกลาง	เห็นด้วยน้อย	เห็นด้วยน้อยที่สุด
1) ท่านเชื่อว่า ผงปรุงรส เช่น ซุปก้อน ผงชูรส สามารถรับประทานได้ไม่มีผลต่อโรคความดันโลหิตสูง					
2) ท่านเชื่อว่าการเติมน้ำปลาหรือซอส ทำให้ท่านรับประทานอาหารได้มากขึ้น					
3) ท่านเชื่อว่า การดื่มน้ำตามหลังการรับประทานอาหารที่มีรสชาติเค็ม จะช่วยขับโซเดียมออกจากร่างกายได้หมด					
4) ท่านคำนึงถึงรสชาติของอาหารมากกว่าปริมาณของเครื่องปรุงที่ใส่ลงไปในอาหาร					
5) ท่านมักจะรู้สึกไม่ดีและไม่มีความสุข ที่ต้องควบคุมการรับประทานอาหาร รสหวาน มัน เค็ม					
6) หากท่านต้องควบคุมหรือลดน้ำหนัก ท่านมักจะกังวลว่าจะไม่ได้กินอาหารที่ชอบหรืออยากกิน					
7) ท่านเชื่อว่าผู้ป่วยโรคความดันโลหิตสูงควรรับประทานอาหารรสจืด					
8) ท่านไม่จำเป็นต้องปรับเปลี่ยนการรับประทานอาหารรสเค็ม เนื่องจากท่านรับประทานยาลดความดันโลหิตสูงอยู่แล้ว					
9) ท่านคิดว่าตัวเองมีพฤติกรรมเสี่ยงต่อการเกิดโรคไตวาย					
10) ท่านมีเป้าหมายในการปรับเปลี่ยนพฤติกรรมเพื่อสุขภาพที่ดี					
11) ท่านมีแรงจูงใจในการปรับเปลี่ยนพฤติกรรมสุขภาพ					
12) ท่านคิดว่าคนในครอบครัวมีส่วนช่วยส่งเสริมสนับสนุนให้ท่านปรับเปลี่ยนพฤติกรรมสุขภาพ					
13) ท่านคิดว่าสิ่งแวดล้อมและชุมชน มีส่วนสนับสนุนให้คนในชุมชนปรับเปลี่ยนพฤติกรรมสุขภาพ					

ส่วนที่ 5 แบบสอบถามพฤติกรรมการป้องกันโรค จำนวน 14 ข้อ

คำชี้แจง โปรดทำเครื่องหมาย ✓ ลงในช่องว่าง ที่ตรงกับความเป็นจริงของท่านมากที่สุด ในแต่ละข้อคำถาม และทำให้ครบทุกข้อ โดยคำตอบมี 3 ตัวเลือก คือ

เป็นประจำ หมายถึง ท่านปฏิบัติกิจกรรมในเรื่องนั้นเป็นประจำ สม่ำเสมอ สัปดาห์ละ 5 วันหรือมากกว่านั้น

บางครั้ง หมายถึง ท่านปฏิบัติกิจกรรมในเรื่องนั้นเป็นบางครั้งหรือบางวัน สัปดาห์ละ 2-4 วัน

ไม่เคย หมายถึง ท่านไม่ปฏิบัติกิจกรรมในเรื่องนั้นเลย หรือปฏิบัติน้อย สัปดาห์ละ 1 วัน

พฤติกรรมการป้องกันโรค	การปฏิบัติตน		
	เป็นประจำ	บางครั้ง	ไม่เคย
1) ท่านประกอบอาหารทานเอง หรือทานอาหารที่บ้านปรุงเอง			
2) ครอบครัวของท่าน ประกอบอาหาร โดยใช้เครื่องปรุง ไม่เกิน 1 – 2 ชนิดเท่านั้น ต่อการปรุงอาหาร 1 อย่าง (ผงชูรส, ผงปรุงรส, น้ำปลา.ซอส ถั่วเหลือง, ซอสน้ำมันหอย, เกลือ ฯลฯ)			
3) ท่านตวงปริมาณเครื่องปรุงในการประกอบอาหารเพื่อควบคุมความเค็ม			
4) ท่านทานอาหารหรือผัก/ผลไม้แปรรูป (เช่นลูกชิ้น หมูยอ กุนเชียง ไส้กรอก ปลาร้า ไข่เค็ม ปลาเค็ม ผักดอง ผลไม้ดอง เป็นต้น)			
5) ท่านทานอาหารประเภทที่มีน้ำซุป หรือน้ำแกง (เช่น น้ำซุปก๋วยเตี๋ยว น้ำแกงทุกประเภท น้ำส้มตำ น้ำผัดผัก น้ำยา)			
6) ท่านทานเนื้อสัตว์ที่มีไขมันสูง กะทิและอาหารทอด เช่น หนังไก่ เครื่องในสัตว์ หอย ปลาหมึก ขาหมูติดมัน หมูสามชั้นและอื่นๆ			
7) ท่านดื่มเครื่องดื่มที่มีน้ำตาลสูง เช่น น้ำอัดลม เครื่องดื่มชูกำลัง น้ำผลไม้สำเร็จรูป น้ำหวาน ชาเย็น กาแฟเย็น ชาเขียว หรืออื่น ๆ			
8) ท่านทานอาหารแต่ละมื้อไม่ได้เติมน้ำปลา/ซีอิ๊ว/ซอส ลงไปในอาหารอีก			
9) บนโต๊ะอาหารท่านจะมีถ้วยพริกน้ำปลา วางไว้เสมอ			
10) ท่านทานอาหารนอกบ้าน เช่น อาหารตามสั่ง, แกงถุง ก๋วยเตี๋ยว ฯลฯ			
11) ท่านทานอาหารนอกบ้าน ท่านจะบอกแม่ค้า ไม่ให้ใส่ผงชูรส, ผงปรุงรส			
12) ท่านอ่านฉลากโภชนาการเพื่อดูชนิดและปริมาณสารอาหารก่อนซื้อ			
13) ท่านออกกำลังกายหรือมีการเคลื่อนไหวร่างกาย เช่น เดิน วิ่ง ปั่นจักรยาน ทำงานบ้าน ทำสวน ทำไร่ จนรู้สึกเหนื่อย สะสมอย่างน้อย			
14) ท่านกินสมุนไพร เช่น ปอกะบิด หญ้าหนวดแมว แห้ม มะรุมแคปซูล เถาวัลย์เปรียง เห็ดหลินจือ รางจืด			
