# Supplementary material for: Community-based intervention for monitoring of salt intake in hypertensive patients: A cluster randomized controlled trial
Source: PLoS One. 2024 Nov 22;19(11):e0311908. doi: 10.1371/journal.pone.0311908 (PMC11584128; doi:10.1371/journal.pone.0311908)
Supplement: S2 Questionnaire — In English. (PDF) [file pone.0311908.s003.pdf]

Questionnaire
Topic: Randomized Cluster Controlled Trial on Salt Consumption and Blood Pressure among Hypertensive Patients in the Community

Instructions before answering the questionnaire:

This questionnaire is used to develop a community salt reduction model, reduce non-communicable diseases in the northern region. It consists of 46 questions divided into 5 parts as follows:

Part 1: General Information Questionnaire (5 questions)
Part 2: Physical Examination Results (4 questions)
Part 3: Assessment of Knowledge and Perception of the Severity of Non-Communicable Diseases (10 questions)
Part 4: Attitude Questionnaire Regarding Behaviors Related to Diabetes, Hypertension, and Chronic Kidney Disease (13 questions)
Part 5: Disease Prevention Behavior Questionnaire (14 questions)

Please kindly answer all questions in the questionnaire. All information provided will be kept confidential by the researchers. The data collected will be summarized and presented for academic purposes only.

Thank you for your cooperation.

Cluster Randomized Controlled Trial on Sodium Salt Consumption and Blood Pressure among Hypertensive Patients in the Community

Part 1: General Information

Instructions: Please mark ✓ in the ☐ or fill in the blank space with the appropriate response according to your situation.

1. Highest level of education:
   - ☐ 1. No formal education, unable to read and write
   - ☐ 2. No formal education, but can read and write
   - ☐ 3. Primary school
   - ☐ 4. Lower secondary education
   - ☐ 5. Upper secondary education/diploma
   - ☐ 6. Bachelor's degree or equivalent
   - ☐ 7. Postgraduate degree or higher
2. Your main occupation (select only one option):
   - ☐ 1. Maid
   - ☐ 2. Farmer (field, garden, rice cultivation)
   - ☐ 3. Merchant/trader
   - ☐ 4. Civil servant
   - ☐ 5. Wage laborer
   - ☐ 6. Other, please specify: _______________
3. Do you have high blood pressure and any other diseases? (You can select more than one option)
   - ☐ 1. Diabetes
   - ☐ 2. High cholesterol
   - ☐ 3. Heart disease
   - ☐ 4. Chronic kidney disease (excluding end-stage)
   - ☐ 5. Stroke
   - ☐ 6. Chronic obstructive pulmonary disease (COPD)
   - ☐ 7. Other, please specify: _______________
   - ☐ 8. None
4. In the past month, have you consumed alcohol?
   - ☐ 1. Yes
   - ☐ 2. No
   - ☐ 3. Used to drink but quit _______ years ago
5. Do you smoke cigarettes?
   - ☐ 1. Yes
   - ☐ 2. No
   - ☐ 3. Used to smoke but quit _______ years ago

Part 2: Physical Examination Results

Instructions: Please mark ✓ in the ☐ or fill in the blank space with the appropriate response according to your actual measurements on the day of assessment.

1. Body weight: __________ kilograms
2. Height: __________ centimeters
3. Waist circumference: __________ centimeters
4. Blood pressure:

1st time………………/………………mmHg
2nd time………………/………………mmHg
3rd time………………/………………mmHg

Part 3: Assessment of Knowledge and Perception of the Severity of Non-Communicable Diseases
Instructions: Please mark ✓ in the blank space that best represents your true knowledge and perception for each question and ensure to answer all questions.

Message	Yes	No	Not sure
1. Salt contains sodium as a component. The body needs only 2,000 milligrams of sodium per day, equivalent to just one teaspoon of salt.			
2. Thai people consume sodium at levels twice what the body needs, which is a significant cause of non-communicable diseases, especially hypertension, kidney disease, heart disease, and stroke.			
3. High-sodium food sources include seasonings (fish sauce, soy sauce, salt, oyster sauce, seasoning powder, soup cubes, and soy dipping sauce) and processed or preserved foods (sausages, meatballs, canned foods, pickled vegetables, pickled fruits, shrimp paste, fermented fish, soybean paste, and fish sauce)			
4. Natural foods inherently contain sodium, whether it's rice, fruits, or meat, etc.			
5. Food labels attached to packaging provide important information about the food composition and key nutrients, including sodium, sugar, fats, etc., which we should review before purchasing.			
6. People who are obese but not genetically predisposed to hypertension and diabetes are not at risk of developing high blood pressure and diabetes.			
7. Hypertension and diabetes are major causes of kidney disease.			
8. Consuming salt increases the rate of waste filtration through the kidneys, making the kidneys stronger.			
9. Adding sweetness to food helps counteract the salty taste, reducing the amount of sodium in the food.			
10. Weight loss, adequate vegetable consumption, and regular exercise do not directly reduce blood pressure levels.			

Part 4: Attitude questionnaire regarding behaviors related to diabetes, hypertension, and kidney disease

Instructions: Please mark ✓ in the blank that corresponds to your most accurate truth for each question and ensure that you complete all questions.

Perception of the benefits and barriers of disease prevention behaviors	Levels				
	Strongly Agree	Agree	Neutral	Disagree	Strongly Disagree
1. You believe that seasoning powders such as bouillon cubes and seasoning powder do not affect high blood pressure.					
2. You believe that adding fish sauce or any type of seasonings makes you eat more.					
3. You believe that drinking water after consuming salty foods help remove sodium from the body completely.					
4. You care more about the true of the food than the amount of seasoning added to it.					
5. You often feel uneasy and unhappy about controlling your intake of sweet, oily, and salty foods.					
6. If you need to control or lose weight, you often worry that you won't be able to eat the food you like or want.					
7. You believe that patients with high blood pressure should eat bland food.					
8. You don't need to change your salty eating habits because you are already taking high blood pressure medication.					
9. You think you have behaviors that are risky for kidney disease.					
10. You have goals to change your behavior for good health.					
11. You are motivated to change your health behaviors.					
12. You think that family members play a role in promoting support to change health behaviors.					
13. You think the environment and community support people in the community to change health behaviors.					

Part 5: Questionnaire on Disease Prevention Behaviors, 14 questions

Instructions: Please mark ✓ in the blank that corresponds to your most accurate truth for each question and ensure that you complete all questions. Each question has three answer options:

Regularly: Indicates that you engage in the activity regularly, consistently, 5 days a week or more.

Sometimes: Indicates that you engage in the activity occasionally or on some days, 2-4 days a week.

Never: Indicates that you never engage in the activity or rarely, only 1 day a week.

Preventive Health Behavior	Behavior		
	Regularly	Sometimes	Never
1. You cook your own meals or eat home-cooked food.			
2. Your family cooks using no more than 1-2 types of seasoning per dish (such as seasoning powder, bouillon cubes, fish sauce, soy sauce, oyster sauce, salt, etc.).			
3. You measure the amount of seasoning in cooking to control saltiness.			
4. You consume processed foods or preserved fruits/vegetables (such as meatballs, pork jerky, fermented pork sausage, fermented fish, salted eggs, salted fish, pickled vegetables, pickled fruits, etc.).			
5. You consume soupy or curry-based dishes (such as noodle soups, curry soups of all types, papaya salad, stir-fried vegetables, dipping sauces).			
6. You consume high-fat animal meats, coconut milk, and fried foods (such as chicken skin, animal organs, shellfish, squid, fatty pork, and others).			
7. You drink sugary beverages (such as soda, energy drinks, packaged fruit juices, sweetened water, iced tea, iced coffee, green tea, or others).			
8. You don't add fish sauce/soy sauce/sauce to your meals anymore.			
9. There is always a bowl of fish sauce on the table.			
10. You eat food from outside, such as take-out, bagged curries, noodles, etc.			
11. When eating out, you ask the vendors not to add seasoning powder or seasoning powder.			
12. You read nutrition labels to check the types and amounts of nutrients before buying.			
13. You exercise or engage in physical activity, such as walking, running, cycling, doing housework, gardening, farming, until you feel tired, accumulating at least.			
14. You consume herbal supplements, such as East Indian screw tree, Kidney tree, Moringa, Lingzhi and laurel clock.			
