# Supplementary material for: Community-based intervention for monitoring of salt intake in hypertensive patients: A cluster randomized controlled trial
Source: PLoS One. 2024 Nov 22;19(11):e0311908. doi: 10.1371/journal.pone.0311908 (PMC11584128; doi:10.1371/journal.pone.0311908)
Supplement: S1 Protocol — (PDF) [file pone.0311908.s004.pdf]

แบบเสนอโครงการวิจัยเพื่อรับการพิจารณาจากคณะกรรมการจริยธรรมการวิจัยในคน
คณะแพทยศาสตร์โรงพยาบาลรามาธิบดี มหาวิทยาลัยมหิดล (ฉบับเต็ม)

1. ชื่อโครงการ

(ไทย) การทดลองควบคุมแบบสุ่มแบบคลัสเตอร์ต่อการบริโภคเกลือโซเดียมและความดันโลหิตในผู้ป่วยโรคความดันโลหิตสูงในชุมชน

(อังกฤษ) Community-based intervention for monitoring of salt intake in hypertensive patients: A Cluster Randomized Controlled Trial

2. ชื่อหัวหน้าโครงการวิจัย

(ไทย) พญ.พิชชาภรณ์ โสนุช

(อังกฤษ) Pitchaporn Sonuch, MD

คุณวุฒิ: แพทย์ประจำบ้านต่อยอด ภาควิชาอายุรศาสตร์ หน่วยโรคไต

สถานที่ทำงาน: ภาควิชาอายุรศาสตร์ หน่วยโรคไต คณะแพทยศาสตร์ โรงพยาบาลรามาธิบดี

สถานที่ที่สามารถติดต่อได้: ภาควิชาอายุรศาสตร์ หน่วยโรคไต อาคาร 1 ชั้น 7 คณะแพทยศาสตร์โรงพยาบาลรามาธิบดี มหาวิทยาลัยมหิดล 270 ถนนพระรามที่ 6 แขวงทุ่งพญาไท เขตราชเทวี กรุงเทพมหานคร 10400

โทรศัพท์: 089-469-9294, 091-774-5981

3. ชื่อผู้ร่วมการวิจัย

3.1 (ไทย) รองศาสตราจารย์นายแพทย์ สุรศักดิ์ กันตชูเวสศิริ

(อังกฤษ) Associate Professor Surasak Kantachuvesiri, MD, PhD

คุณวุฒิ: อาจารย์แพทย์ ภาควิชาอายุรศาสตร์ หน่วยโรคไต

สถานที่ทำงาน: ภาควิชาอายุรศาสตร์ หน่วยโรคไต คณะแพทยศาสตร์โรงพยาบาลรามาธิบดี

สถานที่ที่สามารถติดต่อได้: ภาควิชาอายุรศาสตร์ หน่วยโรคไต อาคาร 1 ชั้น 7 คณะแพทยศาสตร์โรงพยาบาลรามาธิบดี มหาวิทยาลัยมหิดล 270 ถนนพระรามที่ 6 แขวงทุ่งพญาไท เขตราชเทวี กรุงเทพมหานคร 10400

โทรศัพท์: 081-826-7662, 02-201-1301

3.2 (ไทย) ศาสตราจารย์นายแพทย์ วิชัย เอกพลากร

(อังกฤษ) Professor Wichai Aekplakorn, MD, PhD

คุณวุฒิ: อาจารย์แพทย์ ภาควิชาเวชศาสตร์ชุมชน
สถานที่ทำงาน: ภาควิชาเวชศาสตร์ชุมชน คณะแพทยศาสตร์โรงพยาบาลรามาธิบดี
สถานที่ที่สามารถติดต่อได้: ภาควิชาเวชศาสตร์ชุมชน อาคาร 3 ชั้น 8 คณะแพทยศาสตร์โรงพยาบาลรามาธิบดี มหาวิทยาลัยมหิดล 270 ถนนพระรามที่ 6 แขวงทุ่งพญาไท เขตราชเทวี กรุงเทพมหานคร 10400
โทรศัพท์: 02-201-1518, 02-201-1578

3.3 (ไทย) นางสาวณัฐิดา บุญกาญจน์
(อังกฤษ) Natthida Boonyagarn
ตำแหน่ง: นักวิชาการและนักกำหนดอาหารวิชาชีพ
วุฒิการศึกษา: วิทยาศาสตรมหาบัณฑิต สาขาโภชนาการและการกำหนดอาหาร สถาบันโภชนาการ มหาวิทยาลัยมหิดล
สถานที่ทำงาน: เครือข่ายลดบริโภคเค็ม เลขที่ 2 อาคารเฉลิมพระบารมี 50 ปี ชั้น 4 ซอย เพชรบุรี 47 แขวงบางกะปิ เขตห้วยขวาง กรุงเทพมหานคร 10310
โทรศัพท์: 02-716-6091 ต่อ 105
มือถือ: 081-632-2470

3.4 (ไทย) นางสาวศิริพักตร์ มัฆวาล
(อังกฤษ) Siripak Makkawan
ตำแหน่ง: พยาบาลวิชาชีพชำนาญการ
วุฒิการศึกษา: ปริญญาตรี พยาบาลศาสตรบัณฑิต มหาวิทยาลัยพายัพ
สถานที่ทำงาน : สำนักงานป้องกันควบคุมโรคที่ 3 จังหวัดนครสวรรค์ 516/66 ม.10 ถ.พหลโยธิน ต.นครสวรรค์ตก อ.เมือง จ.นครสวรรค์ 60000
โทรศัพท์: 056-221-822 ต่อ 127
มือถือ: 089-354-6441

3.5 (ไทย) นางสาวสุชาดา ธงชาย
(อังกฤษ) Suchada Thongchai
ตำแหน่ง: นักวิชาการสาธารณสุขปฏิบัติการ
วุฒิการศึกษา: วิทยาศาสตรบัณฑิต สาขาสาธารณสุขศาสตร์ (แขนงอนามัยสิ่งแวดล้อม) มหาวิทยาลัยนเรศวร
สถานที่ทำงาน: สำนักงานป้องกันควบคุมโรคที่ 3 จังหวัดนครสวรรค์ 516/66 ม.10 ถ.พหลโยธิน ต.นครสวรรค์ตก อ.เมือง จ.นครสวรรค์ 60000
โทรศัพท์: 056-221-822 ต่อ 127
มือถือ: 083-737-5165

3.6 (ไทย) นางสาววศินี โตสำราญ
(อังกฤษ) Wasinee Tosamran
ตำแหน่ง: นักวิชาการสาธารณสุขปฏิบัติการ
วุฒิการศึกษา: วิทยาศาสตรบัณฑิต สาขาสาธารณสุขศาสตร์ (แขนงอนามัยสิ่งแวดล้อม) มหาวิทยาลัยนเรศวร
สถานที่ทำงาน : สำนักงานป้องกันควบคุมโรคที่ 3 จังหวัดนครสวรรค์ 516/66 ม.10 ถ.พหลโยธิน ต.นครสวรรค์ตก อ.เมือง จ.นครสวรรค์ 60000
โทรศัพท์: 056-221-822 ต่อ 127
มือถือ: 087-010-5043

3.7 (ไทย) นางสาวอนันตญา ขุนจ่าง
(อังกฤษ) Ananthaya Kunjang
ตำแหน่ง: นักวิชาการสาธารณสุข
วุฒิการศึกษา: วิทยาศาสตรมหาบัณฑิต(โรคติดเชื้อและวิทยาการระบาด) คณะสาธารณสุขศาสตร์ มหาวิทยาลัยมหิดล
สถานที่ทำงาน: เครือข่ายลดบริโภคเค็ม เลขที่ 2 อาคารเฉลิมพระบารมี 50 ปี ชั้น 4 ซอย เพชรบุรี 47 แขวงบางกะปิ เขตห้วยขวาง กรุงเทพมหานคร 10310
โทร: 02-716-6091 ต่อ 107
มือถือ: โทร : 083-037-7454

4. ระบุชื่อแพทย์/ผู้ที่จะดูแลผู้เข้าร่วมโครงการวิจัย(ผู้ยินยอมตนให้ทำวิจัย)และติดต่อได้ 24 ชั่วโมง อย่างน้อย 2 คน
4.1 พญ.พิชชาภรณ์ โสนุช
ที่อยู่(ที่ทำงาน): ภาควิชาอายุรศาสตร์ หน่วยโรคไต อาคาร 1 ชั้น 7 คณะแพทยศาสตร์โรงพยาบาลรามาธิบดี มหาวิทยาลัยมหิดล 270 ถนนพระรามที่ 6 แขวงทุ่งพญาไท เขตราชเทวี กรุงเทพมหานคร 10400
ที่อยู่(บ้าน): 588/683 the saint residences condo ถนนวิภาวดีรังสิต แขวงจอมพล เขตจตุจักร กทม. 10900
โทรศัพท์: 089-469-9294, 091-774-5981

4.2 รองศาสตราจารย์นายแพทย์ สุรศักดิ์ กันตชูเวสศิริ
ที่อยู่(ที่ทำงาน): ภาควิชาอายุรศาสตร์ หน่วยโรคไต อาคาร 1 ชั้น 7 คณะแพทยศาสตร์โรงพยาบาลรามาธิบดี มหาวิทยาลัยมหิดล 270 ถนนพระรามที่ 6 แขวงทุ่งพญาไท เขตราชเทวี กรุงเทพมหานคร 10400
โทรศัพท์: 081-826-7662, 02-201-1301
ที่อยู่(บ้าน): 443 ถนนเพชรเกษม แขวงบางหว้า เขตภาษีเจริญ กรุงเทพมหานคร 10160
โทรศัพท์: 081-826-7662

4.3 นางสาวสุชาดา ธงชาย
ที่อยู่(ที่ทำงาน): สำนักงานป้องกันควบคุมโรคที่ 3 จังหวัดนครสวรรค์ 516/66 ม.10 ถ.พหลโยธิน ต.นครสวรรค์ตก อ.เมือง จ.นครสวรรค์ 60000
โทรศัพท์: 056-221822 ต่อ 127
มือถือ: 083-737-5165

5. หลักการและเหตุผล

การได้รับโซเดียมในปริมาณที่สูงเกินความต้องการอย่างต่อเนื่อง ส่งผลต่อการเพิ่มขึ้นของระดับความดันโลหิต เป็นปัจจัยเสี่ยงที่สำคัญในการเกิดโรคหัวใจและหลอดเลือด โรคไต โรคความดันโลหิตสูง และเพิ่มความรุนแรงของโรคเบาหวาน การศึกษาวิจัยพบว่าปริมาณของเกลือโซเดียมที่สูงในอาหารนั้นมีความสัมพันธ์กับค่าความดันโลหิตที่สูง โดยปริมาณเกลือโซเดียมบริโภคที่เพิ่มขึ้น 1 กรัม สามารถเพิ่มระดับความดันโลหิตซิสโตลิก 2.11 มิลลิเมตรปรอท และความดันโลหิตไดแอสโตลิก 0.78 มิลลิเมตรปรอทตามลำดับ(1)

จากสถานการณ์ความรุนแรง และภาระโรค จากโรคไม่ติดต่อ(NCDs) และปัญหาการบริโภคเกลือและโซเดียมในระดับนานาชาติ องค์การอนามัยโลกจึงได้กำหนดให้การลดเกลือโซเดียม เป็น 1 ใน 9 เป้าหมายระดับโลกในการควบคุมปัญหาโรคไม่ติดต่อ (NCDs) ภายใน พ.ศ.2568 โดยกำหนดให้ลดการบริโภคเกลือและโซเดียมลงร้อยละ 30 ภายใน พ.ศ. 2568 ซึ่งประเทศไทยโดยการประชุมสมัชชาสุขภาพแห่งชาติ ครั้งที่ 6 ใน พ.ศ.2557 ได้ทำการรับรองทั้ง 9 เป้าหมายดังกล่าวให้เป็นเป้าหมายในการดำเนินงานของประเทศไทยซึ่งถือเป็นความท้าทายของสังคมไทยในการจัดการปัญหา NCDs อย่างยิ่ง และเพื่อให้เกิดการลดภาระโรคจากโรค NCDs โดยการลดการบริโภคเกลือและโซเดียมในปริมาณสูงในประชากรไทยอย่างจริงจัง จึงได้มีมติสมัชชาสุขภาพแห่งชาติ ครั้งที่ 8 พ.ศ. 2558 เรื่องนโยบายการลดบริโภคเกลือและโซเดียมเพื่อลดโรคไม่ติดต่อ (NCDs) จัดให้มีและขับเคลื่อนยุทธศาสตร์การลดการบริโภคเกลือและโซเดียมในประเทศไทย พ.ศ. 2559-2568 ที่เป็นรูปธรรมต่อไป(2)

จากการสำรวจล่าสุดโดยทีมผู้วิจัยในเครือข่ายลดบริโภคเค็ม พบว่า คนไทยบริโภคเกลือมากถึง 9.1 กรัมต่อวัน ซึ่งสูงเกือบ 2 เท่าของปริมาณที่องค์การอนามัยโลกแนะนำคือไม่เกิน 5 กรัมต่อวัน หรือ 1 ช้อนชา(3) ซึ่งส่วนใหญ่ (71%) ได้รับจากเครื่องปรุงรสเช่น เกลือ น้ำปลา ซีอิ๊ว เต้าเจี้ยว ซุปก้อน ผงชูรส ปลาร้า เป็นต้น และเกลือในอาหารนั้น มากกว่า 60% ละลายอยู่ในน้ำ ไม่ว่าจะเป็นน้ำแกงต่างๆ น้ำซุป น้ำผัด ดังนั้นการลดปริมาณเครื่องปรุง น้ำจิ้มต่างๆ และน้ำซุป จะช่วยลดการบริโภคเกลือได้เป็นอย่างมาก การให้ความรู้และความเข้าใจกับผู้บริโภคในเรื่องของอันตรายจากการบริโภคเค็มว่าส่งผลต่อสุขภาพร่างกายอย่างไร เพื่อค่อยๆ ปรับเปลี่ยนพฤติกรรมการบริโภค จึงจะเป็นวิธีที่จะสามารถเปลี่ยนพฤติกรรมของบริโภคได้อย่างถาวร โดยเฉพาะการปรุงอาหารในบ้าน และแม่ครัวจึงมีความสำคัญอย่างมากในการช่วยให้ผู้บริโภคลดการบริโภคเค็ม อย่างไรก็ตาม การปรับเปลี่ยนพฤติกรรมของผู้บริโภคในเรื่องรสชาติเป็นเรื่องยาก เพราะผู้บริโภคยังคงติดอาหารรสจัดและรสเค็ม และการรับรสเค็มในอาหารนั้นมีความแตกต่างกันในแต่ละคนขึ้นอยู่กับความเคยชิน

สถานการณ์โรคความดันโลหิตสูงในพื้นที่จังหวัดอุทัยธานี พบว่าอัตราป่วยโรคความดันโลหิตสูงต่อประชากรแสนคนตั้งแต่ ปีงบประมาณ 2561-2563 เพิ่มขึ้นอย่างต่อเนื่อง (14,579.01, 150,44.49 และ 17,658.97 ตามลำดับ) ซึ่งอำเภอที่มีการเพิ่มขึ้นของผู้ป่วยสูงเป็นอันดับต้นๆ ของจังหวัดอุทัยธานี คืออำเภอทัพทัน โดยมีอัตราป่วยโรคความดันโลหิตสูงต่อประชากรแสนคนตั้งแต่ ปีงบประมาณ 2561-2563 เพิ่มขึ้นอย่างต่อเนื่อง เท่ากับ 15,280.83, 16,370.88 และ 18,604.19 ตามลำดับ

จากการวิเคราะห์กลไกและกระบวนการจัดการลดการบริโภคเกลือและโซเดียมในประเทศไทย พบข้อจำกัดและความท้าทายในแทบทุกด้าน ตั้งแต่การขาดกลไกการยกระดับการแก้ปัญหาการลดบริโภคเกลือและโซเดียม ไม่มียุทธศาสตร์การดำเนินงานที่ชัดเจน ขาดความเป็นเจ้าของและการมีส่วนร่วมของภาคส่วนต่างๆ ในการจัดการปัญหา รวมถึงกลไกการประสานงานและการบูรณาการผู้เกี่ยวข้อง โดยเฉพาะในภาคส่วนของชุมชน ซึ่งเป็นสิ่งแวดล้อมสำคัญที่ส่งผลต่อรูปแบบวิถีชีวิตของบุคคล โดยเฉพาะการบริโภค และมีผลการศึกษาที่แสดงให้เห็นว่ารูปแบบการบริโภคของบุคคลในชุมชน สอดคล้องเป็นไปตามความนิยมของชุมชนนั้นๆ จึงนำไปสู่การดำเนินงาน “โครงการชุมชนลดเค็ม ลดโรคต้นแบบ ปี 2560” เพื่อศึกษาประสิทธิภาพของมาตรการสำคัญ ที่สามารถสนับสนุนให้ชุมชน สามารถแก้ไขการบริโภคเกลือและโซเดียมในชุมชนได้อย่างมีประสิทธิภาพ

จากการทบทวนมาตรการที่เกี่ยวข้อง พบว่า มาตรการการให้ความรู้เพื่อให้เข้าใจในเรื่องของอันตรายจากการบริโภคเกลือและโซเดียมในปริมาณสูง เป็นวิธีที่จะสามารถปรับเปลี่ยนพฤติกรรมผู้บริโภคได้ อย่างไรก็ดีวิธีการนี้ ค่อนข้างมีความยุ่งยากและขึ้นอยู่กับผู้ให้ความรู้เป็นหลัก โดยถ้าผู้ให้ความรู้สามารถกระตุ้นให้บุคคลเกิดความรู้สึกรักสุขภาพและสามารถปลูกจิตสำนึกให้ลดบริโภคโซเดียมลงได้ ก็จะทำให้ผู้ป่วยได้รับประโยชน์อย่างเต็มประสิทธิภาพ แต่หากผู้ให้ความรู้ขาดประสบการณ์ ขาดเทคนิคในการให้ความรู้ที่จะดึงความสนใจของผู้ป่วยได้ก็จะทำให้การอบรมนั้นไม่ได้ประโยชน์ตามที่คาดหวัง

นอกจากให้ความรู้แล้ว การแสดงให้เห็นปริมาณโซเดียมที่มีอาหาร ด้วยเครื่องวัดความเค็ม หรือ Salt meter ก็ส่งผลต่อความตระหนักได้เป็นอย่างดี คณะผู้วิจัยจึงได้พัฒนาเครื่องมือวัดปริมาณเกลืออย่างง่าย (salt meter) ชนิด PPM/TDS meter โดยร่วมมือกับคณะวิศวกรรมศาสตร์ มหาวิทยาลัยมหิดล โดยใช้หลักการนำไฟฟ้าในสารละลาย สามารถวัดค่าได้ในระดับ Parts Per Million (ppm) และสามารถตั้งค่าให้วัดปริมาณเกลือชนิดต่างๆ โดยเฉพาะเกลือโซเดียมคลอไรด์ เป็นต้น เครื่องนี้ใช้กระแสสลับซึ่งมีความถี่สูงเพื่อให้สามารถวัดค่าความเข้มข้นของเกลือได้ถูกต้อง ตั้งแต่ 0-2% และหน้าจอ ได้พัฒนาให้บุคคลทั่วไปเข้าใจโดยง่ายด้วยการแสดงหน้าจอกราฟฟิครูปหน้าคนในสามระดับ ได้แก่ หน้ายิ้ม จะปรากฏเมื่อความเข้มข้นของเกลืออยู่ในช่วงน้อยกว่า 0.7% แสดงถึงตัวอย่างอาหารมีความปลอดภัยสำหรับรับประทาน หน้าบึ้งปานกลาง จะปรากฏเมื่อความเข้มข้นของเกลือมีค่าอยู่ในช่วง 0.8–0.9% ซึ่งบ่งบอกถึงควรหลีกเลี่ยงอาหารนั้น และหากความเข้มข้นของเกลือมีค่ามากกว่า 1% จะปรากฏหน้าบึ้งมาก บ่งบอกถึงอันตรายไม่ควรรับประทาน

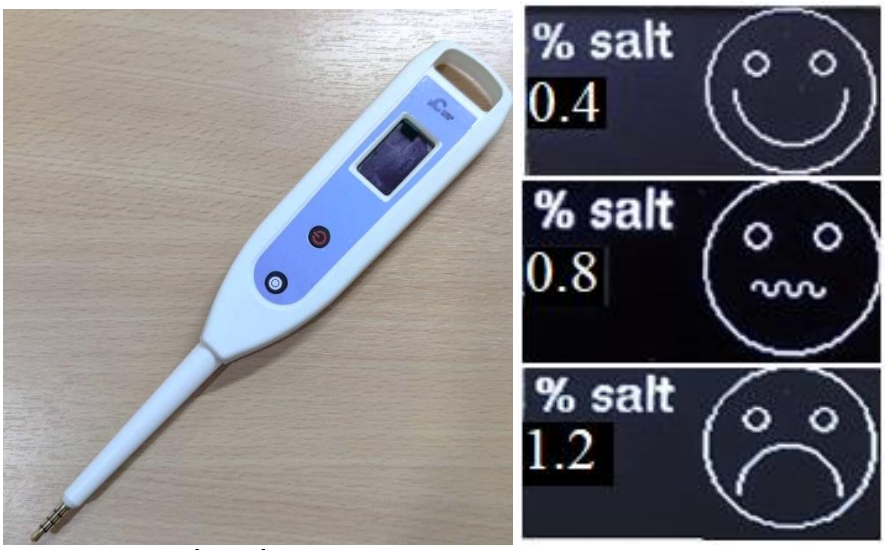

ภาพที่ 1 เครื่องมือวัดปริมาณเกลืออย่างง่าย (salt meter)

จากผลงานวิจัยจากทีมผู้วิจัยในโครงการพัฒนาศักยภาพประชากรไทย คณะแพทยศาสตร์โรงพยาบาลรามาธิบดีใน ปี 2560-62 เรื่อง การใช้เครื่องตรวจวัดโซเดียมคลอไรด์ในอาหาร (salt meter) ด้วยตัวเองต่อพฤติกรรมการบริโภคเกลือโซเดียมและปริมาณโซเดียมในปัสสาวะของผู้ป่วยโรคความดันโลหิตสูง และผลการการทดสอบความไวต่อการรับรสเค็มของผู้ป่วยโรคความดันโลหิตสูงก่อนและหลังการปรับเปลี่ยนพฤติกรรมการบริโภคโซเดียม (งานวิจัยอยู่ระหว่างการตีพิมพ์ของวารสาร Journal of Clinical Hypertension) โดยเป็นการศึกษาแบบสุ่มมีกลุ่มควบคุม ที่หอผู้ป่วยนอก ศูนย์วิจัยคลินิก คณะแพทยศาสตร์โรงพยาบาลรามาธิบดีตั้งแต่วันที่ 1 กันยายน พ.ศ. 2560 ถึง 31 มีนาคม 2563 โดยคัดเลือกคนไข้ความดันโลหิตสูงที่มาตรวจที่หอผู้ป่วยนอกและยังคุมความดันโลหิตไม่ได้ อย่างน้อย 3 เดือน กล่าวคือ ความดันซิสโตลิกมากกว่า 140 มิลลิเมตรปรอทหรือความดันไดแอสโตลิกมากกว่า 90 มิลลิเมตรปรอท โดยสุ่มคนไข้และแบ่งเป็นกลุ่มที่ได้ใช้เครื่องมือวัดปริมาณเกลือในอาหารที่บ้านและกลุ่มควบคุม โดยทั้ง 2 กลุ่มได้รับคำแนะนำเกี่ยวกับการลดการบริโภคเกลือที่ถูกต้องและติดตามผลในระยะที่ 4 และ 8 สัปดาห์ พบว่า คนไข้ทั้งหมด 90 คนที่ผ่านเกณฑ์โดยแบ่งเป็นกลุ่มที่ได้ใช้เครื่องมือวัดปริมาณเกลือในอาหาร (A) 45 คนและกลุ่มควบคุม (B) 45 คน เมื่อสิ้นสุดงานวิจัยที่ 8 สัปดาห์ และติดตามปริมาณเกลือโซเดียม 24 ชั่วโมงในปัสสาวะพบว่ามีการลดลงแตกต่างกันแบบมีนัยสำคัญ คือ 31.83 mmol/24 ชั่วโมง และ 0.36 mmol/24 ชั่วโมง ในกลุ่ม A และกลุ่ม B ตามลำดับ (p=0.006) ค่าเฉลี่ยการลดลงของความดันโลหิตต่างกัน (กลุ่ม A เทียบกับกลุ่ม B) คือ ความดันซิสโตลิกลดลง 14.44 เทียบกับ 8.22 มิลลิเมตรปรอท แบบมีนัยสำคัญ (p=0.030) และ ความดันไดแอสโตลิกลดลง 5.53 เทียบกับ 1.93 มิลลิเมตรปรอทแบบมีนัยสำคัญ (p=0.032) ความไวของการรับรสเกลือดีขึ้นในทั้งสองกลุ่ม แต่มากกว่าในกลุ่ม A หลังจากเสร็จสิ้นการศึกษา ซึ่งข้อสรุปคือการมีเครื่องวัดเกลือสำหรับการตรวจสอบปริมาณเกลือด้วยตนเองร่วมกับการให้ความรู้ด้านอาหารนั้นมีประสิทธิภาพเหนือกว่าการให้ความรู้เพียงอย่างเดียวในผู้ป่วยความดันโลหิตสูงและทำให้การควบคุมความดันโลหิตที่ดีขึ้น รวมทั้งเพิ่มความไวของการรับรสเกลือซึ่งจะทำให้มีผลลดการกินเค็มในระยะยาว

นอกจากมาตรการที่เกี่ยวข้องกับการสร้างความรู้ความตระหนักให้บุคคลในชุมชนแล้ว อีกมาตรการสำคัญที่ควรจัดการควบคู่กันไป คือ มาตรการการจัดการสิ่งแวดล้อม ซึ่งเป็นการปรับองคาพยพของชุมชน สร้างค่านิยมลดการบริโภค

เกลือและโซเดียมในท้องถิ่น และมาตรการปรับสูตรอาหาร สำหรับผู้ผลิตอาหารในชุมชน ให้สามารถปรับเปลี่ยนสูตรอาหารให้ลดปริมาณเกลือและโซเดียม ทำให้ผู้บริโภคไม่มีทางเลือกอาหารที่ดีต่อสุขภาวะ

ซึ่งจากประสิทธิภาพของทั้ง 4 มาตรการที่กล่าวมาข้างต้น โครงการนี้ จึงมุ่งเน้นที่จะแสดงให้เห็นถึงประสิทธิผลของชุดมาตรการดังกล่าว ในพื้นที่ใน จ.อุทัยธานี ได้แก่ เทศบาลเมืองอุทัยธานีและตำบลน้ำซึม อำเภอเมือง, ตำบลหนองฉาง อำเภอหนองฉาง, ตำบลเขาปฐวีและตำบลโคกหม้อ อำเภอทัพทัน และตำบลไผ่เขียว อำเภอสว่างอารมณ์ ที่เข้าร่วมใ นการศึกษาครั้งนี้ ว่าสามารถลดความดันโลหิตและสามารถลดการบริโภคโซเดียมได้ ตามบริบทการบริโภคอาหารที่แตกต่างกัน นอกจากนี้ยังเป็นการทดสอบถึงแนวทางและเทคนิคสำคัญที่สามารถขยายผล ต่อยอดในพื้นที่อื่น ในการจัดการปัญหาการบริโภคโซเดียมที่สามารถนำสู่การปฏิบัติได้จริง และไม่ยุ่งยากซับซ้อน ส่งผลต่อการลดโรคและภัยสุขภาพต่อไป

6. วัตถุประสงค์ของโครงการวิจัย

1. เพื่อเปรียบเทียบความดันโลหิตและปริมาณโซเดียมในปัสสาวะของกลุ่มประชากรก่อนและหลังการให้ความรู้และติดตามการลดบริโภคโซเดียม

2. เพื่อเปรียบเทียบความดันโลหิตและปริมาณโซเดียมในปัสสาวะของกลุ่มประชากรที่ได้รับ การให้ความรู้และติดตามการลดบริโภคโซเดียมและกลุ่มควบคุม

7. วิธีวิจัยและแบบแผนการวิจัย ตารางการทำวิจัย

ขั้นตอนการดำเนินกิจกรรม (วิธีการศึกษา/กลุ่มตัวอย่าง/เครื่องมือที่ใช้ในการศึกษา)

1. การประชุมราชการเพื่อพัฒนาชุดมาตรการ (Intervention package) ร่วมกับผู้เชี่ยวชาญและภาคีเครือข่ายร่วมดำเนินงาน เพื่อการลดหวาน มัน เค็ม

2. การประชุมเชิงปฏิบัติการเพื่อถ่ายทอดพัฒนาชุดมาตรการ (Intervention package) และพัฒนาศักยภาพให้ผู้รับผิดชอบ สำหรับ 3 พื้นที่นำร่อง (พื้นที่ปฏิบัติการระดับตำบล)

3. การลงพื้นที่เพื่อสำรวจลักษณะสำคัญของพื้นที่ปฏิบัติพื้นที่นำร่อง

4. การสนับสนุนงบประมาณในการดำเนินงานให้พื้นที่นำร่อง

5. การติดตามประเมินผลประสิทธิภาพการใช้ชุดมาตรการ (Intervention package) สำหรับกลุ่มประชากรให้กับพื้นที่นำร่อง

ขั้นตอนในการศึกษา

อาสาสมัครจะได้รับคำชี้แจงถึงวัตถุประสงค์และการเข้าร่วมโครงการวิจัย โดยผู้ที่ยินยอมเข้าการวิจัย จะได้รับการตรวจความดันโลหิตและปริมาณเกลือในปัสสาวะใน 24 ชั่วโมง

Intervention Group เป็นระยะเวลา 3 เดือนประกอบด้วย

1. กิจกรรมการให้ความรู้เพื่อการลดหวาน มัน เค็ม อย่างเข้มข้น อาสาสมัครจะได้รับความรู้ คำแนะนำเรื่องผลของการบริโภคเค็มและวิธีการบริโภคอาหารที่มีเกลือโซเดียมต่ำจากแพทย์ รวมทั้งได้รับแผ่นพับความรู้เรื่องการบริโภคเกลือโซเดียมในปริมาณที่ต่ำ

2. การปรับสูตรอาหาร (Reformulation) เพื่อลดการบริโภคเกลือและโซเดียม อาสาสมัครจะได้รับความรู้การปรุงอาหารที่ถูกต้อง

3. การใช้เครื่องมือ Salt meter เพื่อเป็นข้อมูลสะท้อนกลับ พร้อมทั้งจดบันทึกจำนวนครั้งที่ใช้เครื่อง salt meter อาสาสมัครจะได้รับการสอนให้ตรวจปริมาณโซเดียมคลอไรด์ในอาหาร อย่างน้อยวันละ 1 ครั้ง

4. การปรับเปลี่ยนสิ่งแวดล้อม เพื่อการลดบริโภคเกลือและโซเดียม (ปรับสูตรอาหารลดเกลือโซเดียมในร้านค้าในชุมชน เช่น ร้านก๋วยเตี๋ยว ร้านอาหารต่างๆ โดยติดป้ายให้ความรู้เรื่องเกลือโซเดียม ชิมก่อนปรุง และรับรองเมนูเค็มน้อย ปรับการวางพวงเครื่องปรุงรสออกจากโต๊ะอาหารไปที่จุดอื่น

ส่วนผู้ป่วยในกลุ่มที่ 2 เป็นกลุ่มควบคุม ซึ่งจะได้รับการแนะนำและรักษาแบบดั้งเดิม โดยทั้ง 2 กลุ่ม จะไม่มีการปรับยาลดความดันโลหิตของผู้ป่วยตลอดระยะการศึกษา ยกเว้นกรณีความดันโลหิต ซิสโตลิกสูงมากกว่า 180 มิลลิเมตรปรอท หรือต่ำกว่า 100 มิลลิเมตรปรอทหรือมีอาการต่างๆที่เกี่ยวข้องกับความดันโลหิตผิดปกติ ข้อมูลทั้งจากทั้งสองกลุ่มนี้จะถูกเก็บรวบรวมในช่วงก่อนเริ่มการวิจัย และจะมีการตรวจวัดความดันโลหิตทุกๆ 1 เดือน และที่สัปดาห์ที่ 12 หลังเริ่มการวิจัย จะมีการตรวจวัดความดันโลหิตและเก็บปัสสาวะ 24 ชั่วโมง

เครื่องมือที่ใช้ในการศึกษา

1. แบบสอบถามเรื่องการประเมินการรับรู้ข้อมูลด้านสุขภาพโรคไม่ติดต่อเรื้อรัง ภาคเหนือ

2. การเก็บปัสสาวะ 24 ชั่วโมง (24 hours sodium excretion) เพื่อหาปริมาณโซเดียมที่ขับออกมาทางปัสสาวะโดยมีขั้นตอนการเก็บสิ่งส่งตรวจดังนี้

1) เก็บปัสสาวะเป็นระยะเวลารวมทั้งหมด 24 ชั่วโมง เช่น ระยะเวลาเริ่มต้นและระยะเวลาสิ้นสุด ตั้งแต่เวลา 7.00 น. ถึง 7.00 น.ของอีกวัน โดยในการศึกษานี้จะไม่ได้จำกัดเวลาเริ่มเก็บและสิ้นสุดเป็น 7.00 น. ถึง 7.00 น. แต่จะขึ้นอยู่กับระยะเวลาการตื่นนอนของแต่ละบุคคล โดยต้องเก็บให้ครบระยะเวลาภายใน 24 ชั่วโมง

2) เวลาเริ่มต้นจะถูกบันทึกตั้งแต่มีการถ่ายปัสสาวะครั้งแรกทิ้งไป และเริ่มเก็บปัสสาวะครั้งต่อไปเรื่อย ๆ จนครบ 24 ชั่วโมง ในการปัสสาวะทุกครั้งจะต้องมีการบันทึกปริมาตรของปัสสาวะทุกครั้ง

3) Plastic containers ที่ไม่ได้ใช้งานจะถูกเก็บไว้ในตู้เย็น อุณหภูมิ 2-4 °C หรือลังโฟมบรรจุน้ำแข็ง

4) เกณฑ์ที่บ่งบอกปริมาตรที่ถูกต้องของตัวอย่างปัสสาวะ 24 ชั่วโมง คือปริมาตรมากกว่า 500 มิลลิลิตรต่อวัน

5) ในทางปฏิบัติมักพบปัญหาของการเก็บปัสสาวะไม่ครบ 24 ชั่วโมง ซึ่งผู้วิจัยสามารถตรวจสอบได้ว่าปัสสาวะที่เก็บนั้นครบ 24 ชั่วโมงหรือไม่ โดยการตรวจสอบค่าอัตราส่วนของโซเดียมต่อครีเอทรีนีนในปัสสาวะ ในผู้หญิง urine volume> 0.5 liter/day หรือ creatinine excretion มากกว่า 720 mg/day ในผู้หญิงและมากกว่า 980 mg/day ในผู้ชาย

6) ผู้เข้าร่วมวิจัยจะถูกสอบถามข้อมูลทั่วไปส่วนบุคคล รวมถึง ประวัติโรคประจำตัว พร้อมกับการเก็บปัสสาวะ เช่น history of disease, hypertension, body mass index, weight, age, sex, race, socioeconomic status (e.g., income or education), smoking, physical activity, current medication เป็นต้น

3. การวัดระดับความดันโลหิต ด้วยเครื่องดิจิตอล (ที่ผ่านการสอบเทียบมาตรฐาน) มีขั้นตอนดังนี้

1) เตรียมร่างกายให้อยู่ในสภาวะแวดล้อมที่ผ่อนคลาย ก่อนทำการวัด

2) วัดความดันโลหิตที่แขนซ้าย ซึ่งอยู่ใกล้หัวใจ โดยให้จุดที่รับสัญญาณ อยู่ในระดับหัวใจ

3) สวมปลอกแขนที่บริเวณต้นแขน ให้จุดรับสัญญาณอยู่ตรงกลางท้องแขนด้านใน เหนือข้อพับประมาณ 2 - 3 ซม.

4) ติดเทปที่ปลอกแขนให้พอดีกับขนาดแขน ไม่แน่น หรือหลวมจนเกินไป

5) หงายต้นแขนขึ้น แล้ววางแขนบนโต๊ะให้รู้สึกสบาย โดยปลอกแขนจะอยู่ในระดับเดียวกับหัวใจ

6) การวัดความดันโลหิต และควรพักประมาณ 5 นาที ก่อนจะวัด 3 ครั้งใน 5 นาที และใช้ค่าเฉลี่ยของความดันโลหิตครั้งที่ 2 และ 3

ข้อมูลจากแบบสอบถาม

1. อายุและเพศ
2. ระดับการศึกษา
3. อาชีพ
4. ประวัติของโรคความดันโลหิตสูงและโรคประจำตัวอื่นๆ
5. ประวัติการดื่ม alcohol และการสูบบุหรี่

ข้อมูลจากการตรวจร่างกาย

1. ส่วนสูง,น้ำหนัก,ดัชนีมวลกาย, เส้นรอบเอว
2. ความดันโลหิต

แบบประเมินความรู้ และการรับรู้ในความรุนแรงเกี่ยวกับเกี่ยวกับโรคไม่ติดต่อเรื้อรัง

แบบสอบถามทัศนคติเกี่ยวกับพฤติกรรมที่เกี่ยวข้องกับ โรคเบาหวาน โรคความดันโลหิตสูง และโรคไตวาย

แบบสอบถามพฤติกรรมการป้องกันโรค

การวิเคราะห์ข้อมูล

การวิเคราะห์ข้อมูลด้วยสถิติค่าเฉลี่ย(mean) ส่วนเบี่ยงเบนมาตรฐาน(SD) และค่ามัธยฐาน(median) สำหรับข้อมูลเชิงปริมาณ และ 95% confidence intervals (95%CI) ทดสอบการแจกแจงแบบปกติด้วยสถิติ Kolmogorov-Smirnov test วิเคราะห์ความสัมพันธ์ระหว่าง ปริมาณโซเดียมในปัสสาวะและการรับรู้ข้อมูลด้านสุขภาพโรคไม่ติดต่อเรื้อรัง โดยใช้ the Pearson (r) or Spearman (rs) correlation coefficient

8. Protocol Flow Chart

เกณฑ์ในการคัดเข้ามาศึกษา (Inclusion Criteria)

1. บุคคลอายุระหว่าง 18-70 ปี systolic Blood pressure สูง> 130 mmHg
2. ยินดีเข้าร่วมโครงการวิจัยและลงนามในหนังสือยินยอมเข้าร่วมโครงการวิจัย

เกณฑ์ในการคัดออกจากการศึกษา (Exclusion criteria)

1. บุคคลที่มีประวัติเป็นโรคไตวายระยะสุดท้าย
2. บุคคลที่มีประวัติรับประทานยาขับปัสสาวะหรือยาลดความดันโลหิต ที่เพิ่งมีการปรับยาในช่วง2สัปดาห์ที่ผ่านมา
3. หญิงตั้งครรภ์หรือให้นมบุตร
4. บุคคลที่ได้รับเกลือเสริมเนื่องจากเป็นโรคขาดฮอร์โมนสเตียรอยด์หรือ ไตกักเก็บเกลือไว้ไม่ได้หรือความดันโลหิตต่ำ
5. มีการปรับยาความดันโลหิตระหว่างงานวิจัย
6. ปริมาณโซเดียมในปัสสาวะน้อยกว่ากว่า 90 mmol ต่อวันก่อนเข้าการศึกษา
7. ผู้เข้าร่วมวิจัยปฏิเสธหรือขอถอนตัว

↓

เก็บข้อมูลก่อน Intervention ประกอบด้วย

1. วัดระดับความดันโลหิต
2. เก็บตัวอย่างปัสสาวะเพื่อวัดระดับโซเดียมโดยการเก็บปัสสาวะ 24 ชั่วโมง โดยเริ่มเก็บตั้งแต่การครั้งที่ 2 หลังจากตื่นนอน (second void spot fasting morning urine) จนครบเวลา 24 ชั่วโมง
3. ตอบแบบสอบถามข้อมูลทั่วไปส่วนบุคคล ประวัติโรคประจำตัว และแบบสอบถามเรื่องการประเมินการรับรู้ข้อมูลด้านสุขภาพโรคไม่ติดต่อเรื้อรัง ภาคเหนือ (Pre-test)

↓

Intervention Group เป็นระยะเวลา 3 เดือน ประกอบด้วย

1. กิจกรรมการให้ความรู้เพื่อการลดหวาน มัน เค็ม อย่างเข้มข้น
2. การปรับสูตรอาหาร (Reformulation) เพื่อลดการบริโภคเกลือและโซเดียม
3. การใช้เครื่องมือ Salt meter เพื่อเป็นข้อมูลสะท้อนกลับ พร้อมทั้งจดบันทึกจำนวนครั้งที่ใช้เครื่อง salt meter
4. การปรับเปลี่ยนสิ่งแวดล้อม เพื่อการลดบริโภคเกลือและโซเดียม

Control Group เป็นระยะเวลา 3 เดือน

ได้รับคำแนะนำและการรักษาแบบมาตรฐานเดิม

เก็บข้อมูลหลัง Intervention ประกอบด้วย

1. วัดระดับความดันโลหิต โดยมีการติดตามทุกๆ 1 เดือน
2. เก็บตัวอย่างปัสสาวะเพื่อวัดระดับโซเดียมโดยการเก็บปัสสาวะ 24 ชั่วโมง โดยเริ่มเก็บตั้งแต่การครั้งที่ 2 หลังจากตื่นนอน (second void spot fasting morning urine) จนครบเวลา 24 ชั่วโมง โดยมีการติดตามที่ 12 สัปดาห์หลังเข้าร่วมการศึกษา
3. ตอบแบบสอบถามข้อมูลทั่วไปส่วนบุคคล ประวัติโรคประจำตัว และแบบสอบถามเรื่องการประเมินการรับรู้ข้อมูลด้านสุขภาพโรคไม่ติดต่อเรื้อรัง ภาคเหนือ (Post-test)

วิเคราะห์ข้อมูลและสรุปผล

9. ระบุจำนวนผู้เข้าร่วมการวิจัย (Subject) ที่จะศึกษา พร้อมทั้งเกณฑ์คัดเข้าและเกณฑ์คัดออก

การกำหนดกลุ่มตัวอย่าง

ประเมินจำนวนผู้เข้าร่วมวิจัยที่จะได้รับเครื่องตรวจโซเดียมคลอไรด์ในอาหารไว้จำนวน 120 คน และกลุ่มควบคุมอีก 120 คน งานวิจัยนี้สามารถแยกความแตกต่างระหว่าง 2 กลุ่มที่เข้าร่วมวิจัยถึง ร้อยละ 99 ในทั้ง 2 ผลลัพธ์หลักตลอดระยะเวลาการวิจัย 12 สัปดาห์ ผลลัพธ์หลักอันแรก คือ การเปลี่ยนแปลงของปริมาณเกลือโซเดียมในปัสสาวะ 24 ชั่วโมง ให้ได้ร้อยละ 15 นั้น คำนวณโดยใช้การทดสอบแบบสองทางด้วยสถิติ T โดยให้ระดับนัยสำคัญทางสถิติเท่ากับร้อยละ 5 รวมกับจำนวนผู้เข้าร่วมวิจัยที่ขาดการติดตามประเมินไว้ร้อยละ 20 ดังนั้น จำนวนผู้เข้าร่วมวิจัยจึงเพิ่มเป็นกลุ่มละ 60 คน

ขนาดตัวอย่าง

สูตร

$$n= (Za + Zb)^2 (SDi2 + SDc2)/ (mi – mc)^2 * design effect$$

ค่า mi และ m [illegible] ลองและกลุ่มควบคุม เท่ากับ 5 มม.ปรอทในกลุ่ม ท [illegible] -1)rho) โดย กำหนดให้ m คือ cluster size =20 คน แ [illegible]

ขนาดตัวอย่างที่คำนวณได้เท่ากับ 101 คนต่อกลุ่ม เมื่อเผื่อคนที่อาจออกจากการศึกษาก่อนกำหนด กลุ่มละ 120 คน โดยแต่ละกลุ่มมี 6 clusters และจำนวนอาสาสมัครที่เข้าร่วมในแต่ละcluster = 20 คน

ในทางปฏิบัติมีจำนวน รพสต. ที่ในเป็นพื้นที่ศึกษา จ.อุทัยธานี 6 แห่ง ได้แก่ เทศบาลเมืองอุทัยธานีและตำบลน้ำซึม อำเภอเมือง, ตำบลหนองฉาง อำเภอหนองฉาง, ตำบลเขาปฐวีและตำบลโคกหม้อ อำเภอทัพทัน และตำบลไผ่เขียว

อำเภอสว่างอารมณ์ แต่ละพื้นที่ มี 2 กลุ่มที่เข้าร่วมโครงการ รวมเป็น 12 กลุ่ม แบ่งเป็น กลุ่มทดลอง 6 กลุ่ม และกลุ่มควบคุม 6 กลุ่ม

เกณฑ์การคัดเข้า

1. บุคคลอายุระหว่าง 18-70 ปีขึ้นไปและ ความดันโลหิตสูง
2. ยินดีเข้าร่วมโครงการวิจัยและลงนามในหนังสือยินยอมเข้าร่วมโครงการวิจัย

เกณฑ์การคัดออก

1. บุคคลที่มีประวัติเป็นโรคไตวายระยะสุดท้าย
2. บุคคลที่มีประวัติรับประทานยาขับปัสสาวะหรือยาลดความดันโลหิต ที่เพิ่งมีการปรับยาในช่วง2สัปดาห์ที่ผ่านมา
3. หญิงตั้งครรภ์หรือให้นมบุตร
4. บุคคลที่ได้รับเกลือเสริมเนื่องจากเป็นโรคขาดฮอร์โมนสเตียรอยด์หรือ ไตกักเก็บเกลือไว้ไม่ได้หรือความดันโลหิตต่ำ
5. มีการปรับยาความดันโลหิตระหว่างงานวิจัย
6. ปริมาณโซเดียมในปัสสาวะน้อยกว่ากว่า 90 mmol ต่อวันก่อนเข้าการศึกษา
7. ผู้เข้าร่วมวิจัยปฏิเสธหรือขอถอนตัว

10. ระยะเวลาในการศึกษา(ต้องเริ่มหลังจากได้รับการอนุมัติจากคณะกรรมการจริยธรรมการวิจัยในคนแล้ว)

หลังได้รับการอนุมัติจากคณะกรรมการจริยธรรมการวิจัยในคน เป็นระยะเวลา 2 ปี

11. ความเสี่ยงหรือความไม่สบายที่คาดว่าจะเกิดขึ้นกับผู้เข้าร่วมการวิจัย

อาจจะมีความลำบากในการเก็บปัสสาวะ เนื่องจากต้องเก็บ 24 ชั่วโมงตลอดทั้งวัน

12. ประโยชน์ที่คาดว่าจะได้รับ

1. ทราบปริมาณโซเดียมที่ขับออกในปัสสาวะของกลุ่มประชากรเป้าหมาย
2. ความสัมพันธ์ระหว่างพฤติกรรมและความรู้เกี่ยวกับโซเดียมและปริมาณโซเดียมที่พบในปัสสาวะ
3. พฤติกรรมการบริโภคอาหารของกลุ่มประชากร

13. ข้อพิจารณาด้านจริยธรรม

การศึกษาครั้งนี้ ไม่มีการเปิดเผยชื่อจากตัวอย่างปัสสาวะและข้อมูลที่เก็บ โดยผู้วิจัยเก็บข้อมูล และตัวอย่างปัสสาวะจากผู้เข้าร่วมการวิจัยระหว่างการดำเนินการวิจัยเพื่อนำมาวิเคราะห์และประเมินปริมาณโซเดียมที่ขับออกมาทางปัสสาวะ

14. ค่าชดเชยแก่ผู้เข้าร่วมการวิจัย(ตามความจำเป็นและเหมาะสม) ในกรณีที่เกิดอันตรายหรือผลอันไม่พึงประสงค์ต่อผู้เข้าร่วมการวิจัยนี้ อาสาสมัครจะได้รับการดูแลรักษาโดยไม่ต้องเสียค่าใช้จ่ายอย่างใดบ้าง

ผู้เข้าร่วมวิจัยทุกคนจะได้รับเงินค่าตอบแทนเป็นจำนวน 1000 บาทต่อคนตลอดทั้งโครงการ

15. ระบุแหล่งทุนสนับสนุน ในกรณีที่ได้รับทุนสนับสนุนจากภาคเอกชนให้แจกแจงรายละเอียดของงบประมาณและใส่ชื่อผู้ประสานงานของผู้ให้ทุน พร้อมเบอร์โทรศัพท์ที่สามารถติดต่อได้

1. องค์การอนามัยโลก
2. สำนักงานกองทุนสนับสนุนการสร้างเสริมสุขภาพ (สสส.)

รายละเอียดของงบประมาณที่ใช้ในการศึกษาครั้งนี้

รายการ	หน่วย	จำนวน	อัตรา (บาท/หน่วย)	รวม
**งบดำเนินงาน**				
**1. หมวดค่าตอบแทน**				
**1.1** ค่าตอบแทนหัวหน้านักวิจัยในการจัดการความรู้ ผลงานวิจัย การรวบรวมข้อมูล สืบค้นข้อมูล การวิเคราะห์ สังเคราะห์ผลงานวิจัย	เดือน	6	5,000	30,000
**2. หมวดค่าใช้สอย/ดำเนินการ**				
**2.1 ค่าใช้จ่ายสิ่งตอบแทนกลุ่มเป้าหมาย**	คน	240	1,000	240,000
**2.2 อาหารว่างและอาหารกลางวันสำหรับกลุ่มเป้าหมาย**				
**- อาหารว่าง**				
**ครั้งที่ 1** ลงพื้นที่เพื่อประสานงานและประชุมชี้แจงโครงการวิจัย<br>- สำหรับทีมบริหารโครงการวิจัย สคร. และทีมเจ้าหน้าที่ รพ.สต.	มื้อ x คน	1 มื้อ x 10 คน	50	500

**ครั้งที่ 2** ลงพื้นที่เพื่ออบรมชี้แจงการเก็บปัสสาวะและแบบสัมภาษณ์แก่<br>- กลุ่มเป้าหมายและสำหรับทีมวิจัย	มื้อ x คน	1 มื้อ x 260 คน	50	13,000
**- อาหารกลางวัน**				
**ครั้งที่ 1** ลงพื้นที่เพื่อประสานงานและประชุมชี้แจงโครงการวิจัย<br>- สำหรับทีมทีมวิจัยและเจ้าหน้าที่ รพ.สต.	มื้อ x คน	1 มื้อ x 10 คน	100	1,000
**ครั้งที่ 2** ลงพื้นที่เพื่ออบรมชี้แจงการเก็บปัสสาวะและแบบสัมภาษณ์แก่<br>- กลุ่มเป้าหมายสำหรับทีมวิจัยและเจ้าหน้าที่ รพ.สต.	มื้อ x คน	1 มื้อ x 260 คน	100	26,000
**2.3 ผู้ช่วยวิจัย เก็บข้อมูลในพื้นที่ เก็บปัสสาวะและแบบสัมภาษณ์ถาม (นวก.สาสุข, อสม.)**	คน	10 คน	500	5,000
**3. ค่าใช้จ่ายทางห้องปฏิบัติการ**				
- ค่าตรวจ Lab (Urine Na, Urine Cr)		2 Lab x 3 ครั้ง x 240 คน	60 (ก่อน)	86,400
- ค่าตรวจ Lab (Urine Na, Urine Cr)		2 Lab x 3 ครั้ง x 240 คน	60 (หลัง)	86,400
- ค่ารถขนส่งสิ่งส่งตรวจ	ครั้ง	4,500	8	36,000
- ค่าภาชนะ(แกลลอน) สำหรับเก็บปัสสาวะ (ขนาด 5 L)	ใบ	240 x 1	35	8,400
- ค่าน้ำแข็ง	ถุง	240	20	4,800
- ค่าเกลือ	ถุง	240	10	2,400
- ค่ากล่องโฟมเก็บความเย็นสำหรับเก็บภาชนะ	ใบ	240	65	15,600
- ค่าเหยือกพลาสติกเก็บปัสสาวะ	ใบ	240	15	3,600

- ค่าถ่ายเอกสารแบบสอบถาม	ชุด	240	10	2,400
- ค่าเข้าเล่มรายงานสรุป	เล่ม	2	500	1,000
**4. ค่าวิเคราะห์ข้อมูลและลงรหัสข้อมูล**				
- ค่าวิเคราะห์ข้อมูลและลงรหัสข้อมูลค่าจ้างลงรหัสข้อมูล	คน	240	50	12,000
**5. ค่าวัสดุสำนักงานและค่าสาธารณูปโภค**				
- ค่าโทรศัพท์ประสานงานตลอดระยะเวลาการทำวิจัย	เดือน	6	500	3,000
**6. เครื่อง Salt Meter**	อัน	50	1,500	75,000
		**งบประมาณที่ขอจากโครงการ**	**รวมทั้งสิ้น**	**652,500**

ผู้ประสานงานโครงการติดต่อเรื่องการขอทุนสนับสนุนโครงการ

- นางสาวนัธิดา บุญกาญจน์
สถานที่ทำงาน: เครือข่ายลดบริโภคเค็ม เลขที่ 2 อาคารเฉลิมพระบารมี 50 ปี ชั้น 4 ซอย เพชรบุรี 47 แขวงบางกะปิ เขตห้วยขวาง กรุงเทพมหานคร 10310
โทรศัพท์: 02-716-6091 ต่อ 105
มือถือ: 081-632-2470

16. เอกสารชี้แจงข้อมูลและคำแนะนำแก่ผู้เข้าร่วมโครงการ(Patient/Participant Information Sheet)

(เอกสารประกอบ4ก)

17. หนังสือยินยอมโดยได้รับการบอกกล่าวและเต็มใจ(Informed Consent Form)

(เอกสารประกอบ4ก)

18. หลักฐานหรือข้อมูลอ้างอิง

1. Mente A, O'Donnell MJ, Rangarajan S, McQueen MJ, Poirier P, Wielgosz A, et al. Association of urinary sodium and potassium excretion with blood pressure. The New England journal of medicine. 2014 Aug 14;371(7):601-11. PubMed PMID: 25119606.

2. กรมควบคุมโรค กระทรวงสาธารณสุข. ยุทธศาสตร์ลดการบริโภคเกลือและโซเดียมในประเทศไทย พ.ศ. 2559 – 2568. สำนักงานกิจการโรงพิมพ์ องค์การสงเคราะห์ทหารผ่านศึก ในพระบรมราชูปถัมภ์. กรุงเทพมหานคร. 2559.

3. Chailimpamontree W, Kantachuvesiri S, Aekplakorn W et al. Estimated dietary sodium intake in Thailand: A nation-wide population survey with 24-hour urine collections. J Clin Hypertens. 2021; 00:1–11.

19. ลายเซ็นหัวหน้าโครงการวิจัย

ลงชื่อ 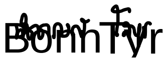 หัวหน้าโครงการ

(พญ.พิชชาภรณ์ โสนุช)

วันที่ 9 ธันวาคม 2564

20. ลายเซ็นอาจารย์ที่ปรึกษา

ลงชื่อ 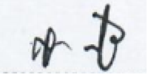 อาจารย์ที่ปรึกษา

(รศ.นพ.สุรศักดิ์ กันตชูเวสศิริ)

วันที่ 9 ธันวาคม 2564
