# Supplementary material for: Community-based intervention for monitoring of salt intake in hypertensive patients: A cluster randomized controlled trial
Source: PLoS One. 2024 Nov 22;19(11):e0311908. doi: 10.1371/journal.pone.0311908 (PMC11584128; doi:10.1371/journal.pone.0311908)
Supplement: S2 Protocol — (PDF) [file pone.0311908.s005.pdf]

Research Proposal for Review by the Research Ethics Committee in Humans

Faculty of Medicine Ramathibodi Hospital, Mahidol University (Full Version)

1. **Project Title**

(Thai)

การทดลองควบคุมแบบสุ่มแบบคลัสเตอร์ต่อการบริโภคเกลือโซเดียมและความดันโลหิตในผู้ป่วยโรคความดันโลหิตสูงในชุมชน

(English) Community-based intervention for monitoring of salt intake in hypertensive patients: A Cluster Randomized Controlled Trial

2. **Project Leader**

(Thai) พญ.พิชชาภรณ์ โสนุช

(English) Pitchaporn Sonuch, MD

Qualifications: General Practitioner, Nephrology Unit, Department of Medicine, Ramathibodi Hospital, Faculty of Medicine, Mahidol University

Workplace: Department of Medicine, Nephrology Unit, Faculty of Medicine, Ramathibodi Hospital, Mahidol University, 270 Rama VI Road, Thung Phaya Thai, Ratchathewi, Bangkok 10400

Contact Information: Department of Medicine, Nephrology Unit, Building 1, 7th Floor, Faculty of Medicine, Ramathibodi Hospital, Mahidol University, 270 Rama VI Road, Thung Phaya Thai, Ratchathewi, Bangkok 10400

Telephone: 089-469-9294, 091-774-5981

3. **Research Collaborator:**

3.1 (Thai) รองศาสตราจารย์นายแพทย์ สุรศักดิ์ กันตชูเวสศิริ

(English) Associate Professor Surasak Kantachuvesiri, MD, PhD

Qualifications: Medical Lecturer, Nephrology Unit, Department of Medicine, Ramathibodi Hospital, Faculty of Medicine, Mahidol University

Workplace: Department of Medicine, Nephrology Unit, Faculty of Medicine, Ramathibodi Hospital, Mahidol University, 270 Rama VI Road, Thung Phaya Thai, Ratchathewi, Bangkok 10400

Contact Information: Department of Medicine, Nephrology Unit, Building 1, 7th Floor, Faculty of Medicine, Ramathibodi Hospital, Mahidol University, 270 Rama VI Road, Thung Phaya Thai, Ratchathewi, Bangkok 10400

Telephone: 081-826-7662, 02-201-1301

3.2 (Thai) ศาสตราจารย์นายแพทย์ วิชัย เอกพลากร
(English) Professor Wichai Aekplakorn, MD, PhD
Qualifications: Medical Lecturer, Department of Community Medicine
Workplace: Department of Community Medicine, Faculty of Medicine Ramathibodi Hospital
Contact Information: Department of Community Medicine, Building 3, 8th Floor, Faculty of Medicine Ramathibodi Hospital, Mahidol University, 270 Rama VI Road, Thung Phaya Thai, Ratchathewi, Bangkok 10400
Telephone: 02-201-1518, 02-201-1578

3.3 (Thai) นางสาวนัธิดา บุญกาญจน์
(English) Natthida Boonyagarn
Position: Nutritionist and Dietitian
Education: Master of Science in Nutrition and Dietetics, Institute of Nutrition, Mahidol University
Workplace: Thai Salt Reduction Network, 2 Chalerm Phrakiat 50th Anniversary Building, 4th Floor, Soi Phetchaburi 47, Bang Kapi, Huai Khwang, Bangkok 10310
Telephone: 02-716-6091 ext. 105
Mobile: 081-632-2470

3.4 (Thai) นางสาวศิริพักตร์ มัฆวาล
(English) Siripak Makkawan
Position: Registered Nurse
Education: Bachelor of Nursing Science, Payap University
Workplace: Department of Disease Control, Nakhon Sawan Province
Address: 516/66 Moo 10, Phaholyothin Road, Nakhon Sawan Takh, Mueang Nakhon Sawan District, Nakhon Sawan 60000
Telephone: 056-221-822 ext. 127
Mobile: 089-354-6441

3.5 (Thai) นางสาวสุชาดา ธงชาย
(English) Suchada Thongchai
Position: Public Health Practitioner
Education: Bachelor of Science in Public Health (Environmental Health), Naresuan University
Workplace: Department of Disease Control, Nakhon Sawan Province
Address: 516/66 Moo 10, Phaholyothin Road, Nakhon Sawan Takh, Mueang Nakhon Sawan District, Nakhon Sawan 60000
Telephone: 056-221-822 ext. 127

Mobile: 083-737-5165

3.6 (Thai) นางสาววศินี โตสำราญ
(English) Wasinee Tosamran
Position: Public Health Practitioner
Education: Bachelor of Science in Public Health (Environmental Health), Naresuan University
Workplace: Department of Disease Control, Nakhon Sawan Province
Address: 516/66 Moo 10, Phaholyothin Road, Nakhon Sawan Takh, Mueang Nakhon Sawan District, Nakhon Sawan 60000
Telephone: 056-221-822 ext. 127
Mobile: 087-010-5043

3.7 (Thai) นางสาวอนันตญา ขุนจ่าง
(English) Ananthaya Kunjang
Position: Public Health Officer
Education: Master of Science (Infectious Diseases and Epidemiology), Faculty of Public Health, Mahidol University
Workplace: Thai Salt Reduction Network, 2 Chalerm Phrakiat 50th Anniversary Building, 4th Floor, Soi Phetchaburi 47, Bang Kapi, Huai Khwang, Bangkok 10310
Telephone: 02-716-6091 ext. 107
Mobile: 083-037-7454

4. Specify the names of the physicians/caretakers overseeing the research participants (consenting to research) and available for contact 24 hours a day. At least 2 individuals.

4.1 Pitchaporn Sonuch, MD
Work Address: Department of Medicine, Nephrology Unit, Building 1, 7th Floor, Faculty of Medicine, Ramathibodi Hospital, Mahidol University, 270 Rama VI Road, Thung Phaya Thai, Ratchathewi, Bangkok 10400
Home Address: 588/683 The Saint Residences Condo, Vibhavadi Rangsit Road, Chom Phon, Chatuchak, Bangkok 10900

Phone: 089-469-9294, 091-774-5981

4.2 Associate Professor Surasak Kantachuvesiri, MD
Work Address: Department of Medicine, Nephrology Unit, Building 1, 7th Floor, Faculty of Medicine, Ramathibodi Hospital, Mahidol University, 270 Rama VI Road, Thung Phaya Thai, Ratchathewi, Bangkok 10400

Phone: 081-826-7662, 02-201-1301
Home Address: 443 Petchkasem Road, Bang Wa, Phasi Charoen, Bangkok 10160
Phone: 081-826-7662

4.3 Suchada Thongchai
Work Address: Department of Disease Control, Nakhon Sawan Province, 516/66 Moo 10, Phaholyothin Road, Nakhon Sawan Takh, Mueang Nakhon Sawan District, Nakhon Sawan 60000
Phone: 056-221822 ext. 127
Mobile: 083-737-5165

5. **Principles and Reasons**

Excessive intake of sodium beyond the body's requirement continuously increases blood pressure levels. It is a significant risk factor for the development of cardiovascular diseases, kidney diseases, hypertension, and exacerbation of diabetes. Research studies have found that high levels of dietary sodium are correlated with high blood pressure levels. An increase of 1 gram of sodium consumption can raise systolic blood pressure by 2.11 millimeters of mercury and diastolic blood pressure by 0.78 millimeters of mercury, respectively.

From the severity of the situation and the burden of disease from non-communicable diseases (NCDs) and the issue of salt and sodium consumption internationally, the World Health Organization has designated the reduction of sodium consumption as one of the nine global targets for controlling non-communicable diseases (NCDs) by 2568 B.E. It is aimed to reduce salt and sodium consumption by 30% by 2568 B.E. In Thailand, during the 6th National Health Assembly in 2557 B.E., they endorsed all nine mentioned goals as targets for Thailand's action, representing a challenge for Thai society in managing NCDs issues. To reduce the burden of disease from NCDs by reducing high salt and sodium consumption among the Thai population, a resolution was made during the 8th National Health Assembly in 2558 B.E. Regarding policies to reduce salt and sodium consumption to control NCDs, the strategies for salt and sodium reduction in Thailand was proposed for 2559-2568 B.E.

According to the latest survey by the salt reduction network research team, Thais consume as much as 9.1 grams of salt per day, which is nearly double the amount recommended by the World Health Organization, which is not more than 5 grams per day or 1 teaspoon. Most of this (71%) comes from seasoning such as salt, fish sauce, soy sauce, oyster sauce, bouillon cubes, seasoning powder, shrimp paste, etc. Moreover, salt in food is mostly dissolved in water, such as in various soups and stir-fries. Therefore, reducing the use of seasonings, dipping sauces, and soup bases can significantly reduce salt consumption. Providing knowledge and understanding to consumers about the dangers of excessive salt consumption and its impact on health is essential for gradually changing consumption behavior, especially in home cooking, where the role of cooks is crucial in helping consumers reduce

salt intake. However, changing consumer behavior regarding taste preferences is challenging because consumers still prefer salty and strongly flavored foods, and the acceptance of saltiness varies among individuals depending on their accustomed taste.

Regarding the situation of hypertension in Uthai Thani province, it was found that the incidence of hypertension per 100,000 population has been continuously increasing from the fiscal year 2561-2563 (14,579.01, 150,44.49, and 17,658.97 respectively), with the districts experiencing the highest increase in patients being among the top in the province. For example, in Thap Than district, the incidence of hypertension per 100,000 population has continuously increased from fiscal year 2561-2563, with figures of 15,280.83, 16,370.88, and 18,604.19 respectively.

From the analysis of mechanisms and processes for reducing salt and sodium consumption in Thailand, limitations and challenges are found in almost every aspect, from lacking mechanisms to elevate the level of addressing salt and sodium reduction issues, lacking clear operational strategies, lacking ownership and involvement of various sectors in addressing the problem, including coordination mechanisms and integration of stakeholders, particularly in the community sector, which is an important environment that affects individuals' lifestyles, especially consumption, and studies have shown that individuals' consumption patterns in the community align with the community's preferences, leading to the implementation of the "Community Salt Reduction Prototype Project 2560" to study the effectiveness of key measures to support communities in effectively addressing salt and sodium consumption.

Reviewing related measures, it is found that providing knowledge to understand the dangers of excessive salt and sodium consumption is a way to change consumer behavior. However, this method is relatively complicated and depends on the knowledge provider. If knowledge providers can stimulate individuals to feel health-conscious and cultivate awareness to reduce sodium consumption effectively, patients will benefit efficiently. Still, if knowledge providers lack experience and techniques to stimulate interest among patients, the training will not be as effective as expected.

Apart from providing knowledge, showing the amount of sodium present in food using a salt meter also contributes to raising awareness effectively. The research team has developed a simple tool (salt meter) in collaboration with the Faculty of Engineering, Mahidol University, using the principle of electricity conductivity in a solution, capable of measuring values in Parts Per Million (ppm) and setting values to measure various types of salts, especially sodium chloride. This device uses alternating currents with high frequency to accurately measure salt concentrations from 0-2%, and the screen is developed to be easily understood by the general public, with graphical representations on three levels: smiling face, indicating safety for consumption when salt concentration is less than 0.7%; neutral face, indicating avoidance of consumption when salt concentration is in the range of 0.8-0.9%; and frowning face, indicating high danger and should be avoided when salt concentration is more than 1%.

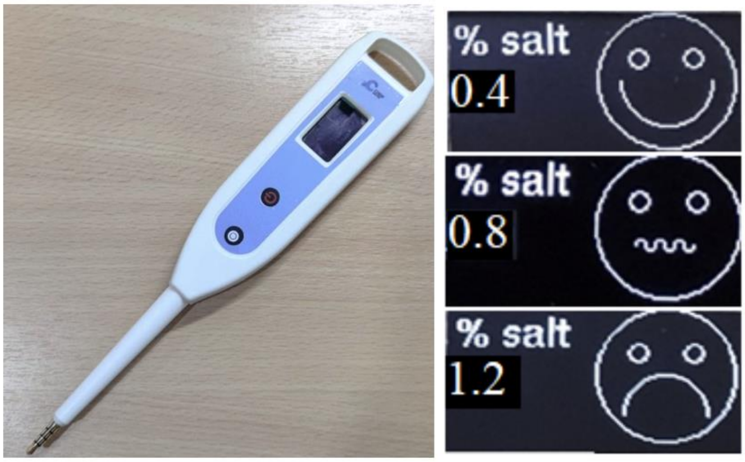

Image 1 Salt Meter - a tool for easily measuring salt quantity

From the research work by the research team in the Thai Population Development Project, Faculty of Medicine, Ramathibodi Hospital, during the years 2560-62, on the topic of using a salt meter to measure sodium chloride in food by oneself to modify sodium consumption behavior and urinary sodium excretion in patients with hypertension. Also, the results of taste sensitivity testing for salt intake in hypertensive patients before and after sodium consumption behavior modification. This research is pending publication in the Journal of Clinical Hypertension.

The study is a randomized controlled trial conducted at the outpatient clinic, Clinical Research Center, Faculty of Medicine, Ramathibodi Hospital, from September 1, 2560, to March 31, 2563. Patients with high blood pressure who had been under blood pressure control for at least 3 months were selected. This means systolic blood pressure above 140 millimeters of mercury (mmHg) or diastolic blood pressure above 90 mmHg. Patients were randomized and divided into a group that used a salt measuring tool at home and a control group. Both groups received advice on proper salt reduction and were followed up at 4 and 8 weeks. It was found that all 90 patients who met the criteria were divided into Group A (those who used the salt measuring tool) with 45 people and Group B (control group) with 45 people. At the end of the 8-week study and follow-up of 24-hour urinary sodium levels, a significant difference in sodium reduction was found: 31.83 mmol/24 hours and 0.36 mmol/24 hours in Group A and Group B respectively ($p=0.006$). The average reduction in blood pressure differed between Group A and Group B: systolic blood pressure decreased by 14.44 compared to 8.22 mmHg, respectively ($p=0.030$), and diastolic blood pressure decreased by 5.53 compared to 1.93 mmHg, respectively ($p=0.032$). Taste sensitivity to salt improved in both groups but more in Group A after the study period.

In conclusion, having a salt measuring device for self-monitoring and nutritional education is more effective than providing education alone for hypertensive patients, leading to better blood pressure control and improved taste sensitivity to salt, which may result in long-term reduction in salt intake.

In addition to measures related to building knowledge and awareness in the community, another important measure to be implemented in parallel is environmental management, which involves adjusting the community's environment to create a preference for reducing salt and sodium consumption locally.

This includes adjusting food recipes for local food producers to reduce salt and sodium content, providing consumers with no option but to choose healthier food options.

Based on the effectiveness of the forementioned four measures, this project focuses on demonstrating the effectiveness of these measures in the areas of Uthai Thani Municipality and Namsuem Subdistrict, Mueang District, Nong Chang Subdistrict, Nong Chang District, Khao Phatthawee Subdistrict, and Kok Mor Subdistrict, Thap Than District, and Phai Khiao Subdistrict, Sawang Arom District, which participated in this study. It shows that it is possible to reduce blood pressure and sodium consumption according to different food consumption contexts. Furthermore, it is a test of important guidelines and techniques that can be expanded to other areas to address the problem of sodium consumption, which can lead to practical and not overly complicated solutions, resulting in the reduction of disease and health risks in the future.

6. Objectives of the Research Project

1. To compare blood pressure and sodium levels in urine among the population group before and after receiving knowledge and follow-up on reducing sodium consumption.
2. To compare blood pressure and sodium levels in urine among the population group receiving knowledge and follow-up on reducing sodium consumption with the control group.

7. Research Methodology and Research Plan: Research Activity Table Research Activity Steps

(Research Methods/Sample Groups/Study Tools Used)

1. Government meetings to develop an Intervention package together with experts and collaborative network sectors to reduce sugar, fat, and salt intake.
2. Operational meetings to transfer the development of an Intervention package and develop capabilities for responsible persons in 3 pilot areas (sub-district level operations).
3. Field visits to survey key characteristics of pilot operational areas.
4. Budget support for operational activities in pilot areas.
5. Monitoring and evaluating the effectiveness of using the Intervention package for the population group in the pilot areas.

Study Procedure

Volunteers will be briefed on the objectives and participation in the research project. Those who consent to participate in the research will undergo blood pressure and urine sodium level tests within 24 hours.

Intervention Group consists of a 3-month period, including:

1. Intensive educational activities for reducing sugar, fat, and salt consumption. Volunteers will receive knowledge and advice on the effects of salt consumption and methods of consuming low-sodium foods from physicians. They will also receive pamphlets on low-sodium salt consumption.
2. Food reformulation to reduce salt and sodium consumption. Volunteers will receive knowledge on proper food preparation.
3. Utilization of Salt meters for feedback data, along with recording the frequency of Salt meter usage. Volunteers will be taught to measure sodium chloride levels in food at least once a day.
4. Environmental changes to reduce salt and sodium consumption (reducing sodium salt in community stores such as noodle shops, and various restaurants, by providing informative signage on sodium salt, encouraging tasting before seasoning, and certifying low-salt menus. Adjusting the placement of seasoning away from dining tables). Patients in Group 2 are the control group, receiving advice and treatment as usual. Throughout the study period, both groups will not have their blood pressure medication adjusted unless their systolic blood pressure is above 180 millimeters of mercury (mm Hg) or below 100 mm Hg, or they experience symptoms related to abnormal blood pressure. Data from both groups will be collected before the study begins, and blood pressure will be measured every month, with urine collected for 24 hours at week 12 after the study begins.

Tools Used in the Study:

1. Questionnaire on the Assessment of Non-Communicable Disease Health Perception, Northern Region

2. Collection of 24-hour Urine (24 hours sodium excretion) to determine the amount of sodium excreted in the urine involves the following steps:

1) The urine is collected over a total period of 24 hours, for example, starting from 7:00 a.m. to 7:00 a.m. of the following day. However, in this study, the start and end times of urine collection are not limited to 7:00 a.m. to 7:00 a.m. It depends on each individual's waking time, but the collection must be completed within 24 hours.
2) The start time is recorded from the first urination, and subsequent urine collections continue until completing 24 hours. The volume of urine must be recorded each time.
3) Plastic containers that are not in use are stored in the refrigerator at 2-4 °C or in a foam box containing ice.
4) The criterion indicating the correct volume of a 24-hour urine sample is a volume greater than 500 milliliters per day.
5) In practice, there is often an issue with incomplete urine collection for 24 hours. Researchers can check whether the collected urine is complete for 24 hours by examining the sodium-to-creatinine ratio in the urine. In females, the urine volume should be greater than 0.5 liter/day or creatinine excretion should be more than 720 mg/day. In males, creatinine excretion should be more than 980 mg/day.

6) Participants will be asked for general personal information, including medical history, hypertension, body mass index, weight, age, sex, race, socioeconomic status (e.g., income or education), smoking, physical activity, and current medication, along with urine collection information.

3. Measuring Blood Pressure Levels with a Digital Device (Standardized):

1) Prepare the body to be in a relaxed environment before taking measurements.
2) Measure blood pressure on the left arm, close to the heart, with the sensor level with the heart.
3) Wear the arm cuff around the upper arm, ensuring the sensor point is approximately 2-3 cm above the elbow crease.
4) Secure the cuff with tape, ensuring it fits snugly but not too tight or loose.
5) Position the arm comfortably on a table with the elbow slightly raised so that the arm cuff is at the same level as the heart.
6) Take blood pressure readings after resting for about 5 minutes. Measure three times within 5 minutes and use the average of the second and third readings.

<u>Information from the questionnaire</u>

1. Age and gender
2. Level of education
3. Occupation
4. History of high blood pressure and other medical conditions
5. History of alcohol consumption and smoking.

<u>Data from Physical Examination</u>

1. Height, weight, body mass index (BMI), waist circumference
2. Blood pressure

<u>Assessment of Knowledge and Perception Regarding Non-Communicable Diseases</u>

<u>Questionnaire on the perception of severity regarding non-communicable diseases</u>

Attitude questionnaire regarding behaviors associated with diabetes, hypertension, and kidney disease

<u>Questionnaire on disease prevention behaviors</u>

Data Analysis

Statistical analysis includes calculating the mean, standard deviation (SD), and median for quantitative data.

Utilize 95% confidence intervals (95% CI).

Test normal distribution using the Kolmogorov-Smirnov test.

Analyze the relationship between urinary sodium levels and perception of non-communicable disease health data using the Pearson (r) or Spearman (rs) correlation coefficient.

8. Protocol Flow Chart

Inclusion Criteria

1. Individuals aged between 18-70 years old with systolic blood pressure > 130 mmHg.
2. Willingness to participate in the research project and signing the informed consent form to join the study.

Exclusion Criteria

1. Individuals with a history of end-stage kidney disease.
2. Individuals with a history of taking diuretics or antihypertensive medication, which has been adjusted within the past 2 weeks.
3. Pregnant or lactating women.
4. Individuals who have received salt supplementation due to hormonal deficiencies or salt retention, and who do not have low blood pressure.
5. Individuals who have had their blood pressure medication adjusted during the study.
6. Urinary sodium levels less than 90 mmol per day before entering the study.
7. Participants who refuse to participate or request withdrawal.

↓

Data Collection Before Intervention Includes:

1. Measuring blood pressure levels.
2. Collecting urine samples to measure sodium levels. Urine collection will be conducted over a 24-hour period, starting from the second void spot in the morning (fasting morning urine), until completing 24 hours.
3. Responding to a questionnaire covering general personal information, medical history, and a pre-test questionnaire assessing perception of non-communicable disease health data in the Northern Region.

↓

Intervention Group for a 3-Month Period Includes

1. Intensive educational activities aimed at reducing sugar, fat, and salt intake.
2. Dietary reformulation to reduce salt and sodium consumption.
3. Utilization of a Salt meter tool to provide feedback, along with recording the frequency of Salt meter usage.

Control Group for a 3-Month Period

Received standard advice and treatment protocol

↓

Data Collection After Intervention Includes

1. Measuring blood pressure levels with monthly follow-ups.
2. Collecting urine samples to measure sodium levels. Urine collection will be conducted over a 24-hour period, starting from the second void spot in the morning (fasting morning urine), until completing 24 hours. Follow-up will be conducted at the 12-week mark after joining the study.
3. Responding to a questionnaire covering general personal information, medical history, and a post-test questionnaire assessing perception of non-communicable disease health data in the Northern Region.

↓

Data analysis and summary

9. Specify the number of research subjects to be studied, along with the inclusion and exclusion criteria.

Sample Group Assignment

Evaluate the number of research participants to receive sodium chloride test kits in food, totaling 120 people, and another control group of 120 people. This study can differentiate between the two participating groups by 99% in both main outcomes throughout the 12-week research period. The first main result is a 15% change in sodium chloride quantity in urine within 24 hours, calculated using a two-way statistical T-test with a statistical significance level of 5%, along with a 20% loss to follow-up in the number of research participants. Therefore, the number of research participants has increased to 60 people per group.

Sample size

Formula:

$$n = (Za + Zb)^2 \, (SDi2 + SDc2) / (mi - mc)^2 * \text{design effect}$$

The values mi and mc represent the changes in systolic blood pressure in the experimental group and the control group, respectively. They are equal to 5 mmHg in the experimental group and 0 in the control group. For the design effect = (1+ (m-1)rho), where m is the cluster size = 20 people, and rho is the intracluster correlation = 0.2.

The calculated sample size is 101 individuals per group, with an allowance for potential dropouts before assignment. Each group will have 120 individuals, divided into 6 clusters with 20 individuals each.

In practice, there are a total of 6 hospitals in the study area of Uthai Thani province, including Uthai Thani municipality and Namsuem sub-district in Muang district, Nong Chang sub-district in Nong Chang district, Khao Phthawi and Kok Mo sub-districts in Thap Than district, and Phai Khiao sub-district in Sawang Arom district. Each area has 2 groups participating in the project, totaling 12 groups divided into 6 experimental groups and 6 control groups.

Inclusion criteria

1. Individuals aged between 18-70 years old with high blood pressure.
2. Willingness to participate in the research project and sign the consent form to join the research project.

Exclusion criteria

1. Individuals with a history of end-stage renal disease.
2. Individuals who have recently had their diuretic or antihypertensive medication adjusted within the past 2 weeks.
3. Pregnant or lactating women.

4. Individuals receiving salt supplementation due to thyroid hormone deficiency or inability to retain salt, or individuals with low blood pressure.
5. Those who have had adjustments to their blood pressure medication during the study.
6. Urinary sodium excretion less than 90 mmol per day prior to enrollment.
7. Participants who refuse to participate in the research or request withdrawal.

10. Study duration (to commence after approval from the research ethics committee)

Following approval from the research ethics committee, the study will last for 2 years.

11. Potential risks or discomforts expected for participants

Difficulty in urine collection as it requires 24-hour collection throughout the day.

12. Expected benefits

1. Understanding the urinary sodium excretion quantity among the target population.
2. The relationship between behavior and knowledge regarding sodium and the amount of sodium found in urine.
3. Dietary consumption behavior of the study population.

13. Ethical considerations

This study will not disclose the names of urine samples and the collected data. Researchers will collect data and urine samples from research participants during the research process for analysis and evaluation of urinary sodium excretion.

14. Compensation for research participants (as necessary and appropriate) In the event of harm or undesirable outcomes, volunteers will receive care without any financial burden.

Each research participant will receive a compensation of 1000 Baht per person throughout the project.

15. Funding source: In case of funding from the private sectors, provide details of the budget and contact information of the funding coordinator, including telephone number.

1. World Health Organization (WHO)
2. Health Promotion Fund (HPF), Department of Health.

Details of the budget used in this study.

List	Unit	Quantity	Rate (baht/unit)	Total
**Operating budget**				
**1. Compensation category**				
1.1 Research Leader Compensation for Knowledge Management, Research Output, Data Collection, Information Retrieval, Analysis, and Research Synthesis.	Month	6	5,000	30,000
**2. Expense/Operation category**				
**2.1 Compensation for Target Group Participants.**	Person	240	1,000	240,000
**2.2 Snacks and Lunch for Target Group Participants.**				
**- Snacks**				
**First time:** field visit for project coordination and research project briefing.<br>- For the research management team, project coordinators, and hospital staff.	Meal x People	1 meal x 10 people	50	500
**Second time:** entering the area for training to explain urine collection and interview forms to<br>- Target groups and research teams.	Meal x People	1 meal x 260 people	50	13,000
**- Lunch**				

List	Unit	Quantity	Rate (baht/unit)	Total
**First time** entering the area to coordinate and conduct a research project briefing:<br>- For the research team and hospital staff.	Meal x People	1 meal x 10 people	100	1,000
**Second time** entering the area to conduct training on urine collection and interviews for:<br>- Target groups for the research team and hospital staff.	Meal x People	1 meal x 260 people	100	26,000
2.3 Research assistants collect data in the field, collect urine samples, and conduct interviews:<br>(Public health officers, medical assistants.)	Person	10 people	500	5,000
**3. Laboratory expenses**				
- Laboratory testing fee (Urine Na, Urine Cr)		2 Lab x 3 times x 240 people	60 (before)	86,400
- Laboratory testing fee (Urine Na, Urine Cr)		2 Lab x 3 times x 240 people	60 (after)	86,400
- Transportation fee for sending samples for testing	Times	4,500	8	36,000
- Container (can) for urine collection (5L size)	Sheets	240 x 1	35	8,400
- Ice fee	Bags	240	20	4,800
- Salt fee	Bags	240	10	2,400
- Foam box fee for container refrigeration	Sheets	240	65	15,600

List	Unit	Quantity	Rate (baht/unit)	Total
- Plastic urine collection cup fee	Sheets	240	15	3,600
- Questionnaire photocopying fee	Sets	240	10	2,400
- Fee for accessing the report summary booklet	Booklets	2	500	1,000
**4. Data analysis and data encoding fee**				
- Data analysis and encoding fee for data encoding service	Person	240	50	12,000
**5. Office supplies and utility fees**				
- Communication expenses (telephone) throughout the research period	Month	6	500	3,000
**6. Salt Meter equipment**	Piece	50	1,500	75,000
		**Budget requested from the project.**	**Total**	**652,500**

Project Coordinator Contact Information for Funding Support:

- Ms. Natthida Boonganjorn

Workplace: Thai Salt Reduction Network, Building 2, Chaloem Phra Baramee 50 Years, 4th Floor, Soi Phetchaburi 47, Bangkapi Sub-district, Huai Khwang District, Bangkok 10310

Phone: 02-716-6091 ext. 105

Mobile: 081-632-2470

16. Documents providing information and guidance to participants (Patient/Participant Information Sheet) (Supplementary Document 4a)

17. Informed Consent Form (Supplementary Document 4b)

18. References:

Mente A, O'Donnell MJ, Rangarajan S, McQueen MJ, Poirier P, Wielgosz A, et al. Association of urinary sodium and potassium excretion with blood pressure. The New England journal of medicine. 2014 Aug 14;371(7):601-11. PubMed PMID: 25119606.

Department of Disease Control, Ministry of Public Health. Salt and Sodium Reduction Strategy in Thailand, B.E. 2559 – 2568. Army Welfare Printing Office, Royal Thai Armed Forces Headquarters. Bangkok. 2559.

Chailimpamontree W, Kantachuvesiri S, Aekplakorn W et al. Estimated dietary sodium intake in Thailand: A nationwide population survey with 24-hour urine collections. J Clin Hypertens. 2021; 00:1–11.

19. Signature of the Research Project Leader

Signed: Pichayaphorn Sonnuch

Date: 9 December 2021

20. Signature of the Advisor Professor

Signed: Assoc. Prof. Dr. Surasak Kuntachuvesiri

Date: 9 December 2021
