# Supplementary material for: Community-based intervention for monitoring of salt intake in hypertensive patients: A cluster randomized controlled trial
Source: PLoS One. 2024 Nov 22;19(11):e0311908. doi: 10.1371/journal.pone.0311908 (PMC11584128; doi:10.1371/journal.pone.0311908)
Supplement: Attachment — Submitted filename: Response to reviewers.docx [file pone.0311908.s007.docx]

**Response to reviewers**

Reviewer #1: The manuscript is quite well written. However, the manuscript could be refined.

Comments
More information on the randomization method and process, and who performed it is to be provided. – completed, lines 96–102 of the tracked changes version .

Ans. Each health center was responsible for 10-12 villages. We used a random number generator, to assign a random number (1, 2) to each village (cluster). The villages assigned the number 1 were the intervention group, and the villages assigned the number 2 were the control group. A total of 6 clusters were randomly allocated to the intervention group and another 6 clusters to the control group. A total of 20 patients meeting the eligible criteria were randomly recruited, using the random number generator, in each cluster. Informed consent was obtained from all participants. (Fig 1)

Line 106, more information on the standard questionnaire on their knowledge, attitude, and behaviour is to be provided e.g. reliability and validation information of the questionnaire in the context of local setting. – completed, lines 109-112.

Ans. The questionnaire was developed according to the local context and its content validity was assessed by 3 experts. It was tested for reliability, with the instrument achieving a Cronbach's alpha coefficient of 0.79 [12].

For sample size calculation, the formula for the sample size and the value corresponding to each item is to be displayed. – completed, lines 135-137.

Ans.

$$\boldsymbol{n=}\frac{\left( \boldsymbol{a+b} \right)^{\boldsymbol{2}}\left( \boldsymbol{SD}_{\boldsymbol{i}}^{\boldsymbol{2}}\boldsymbol{+}\boldsymbol{SD}_{\boldsymbol{c}}^{\boldsymbol{2}} \right)}{\boldsymbol{(}\boldsymbol{m}_{\boldsymbol{i}}\boldsymbol{-}\boldsymbol{m}_{\boldsymbol{c}}\boldsymbol{)}^{\boldsymbol{2}}}$$

a= Z _alpha_=1.96, b= Z_1-beta_=1.28, SD= standard deviation of BP for intervention and control, mi-mc =mean changes of BP between intervention and control.

Line 131-132, for Fisher’s exact test, 1 or 2-tailed test and Line 132, the specific name of the t-test to be stated. – completed, lines 139-141.

Ans. Categorical variables were analyzed using Fisher's exact test when expected values were less than 5, while continuous variables were analyzed using the student’s t-test with 2-tailed test.

Line 133, comparison between groups whether at certain time point(s) between the groups or the differences in pre and post-intervention between the two groups is to be stated. – completed, lines 141-143 .

Ans. The primary outcomes were evaluated using the Wilcoxon rank-sum test, while the Wilcoxon signed-rank test was used to compare urine sodium excretion within group between pre and post intervention and between two groups.

Line 134, the statement ‘The change of blood pressure was performed with linear mixed-effects model’ and Line 135 with the statement ‘In addition, the changes of variables between the group and associated test of effect were estimated by regression’ requires more information. A statement on fulfilment of LMM assumptions is to be stated. – completed, lines 144-146.

Ans. The change of blood pressure was performed with linear mixed-effects model (LMM) since the study measured blood pressure (BP) multiple times on each participant before and after intervention. LMMs account for the correlation between these repeated measures within each participant. We used an unstructured correlation structure.

Line 134, as the change of blood pressure was measured at baseline, 4 weeks, 8 weeks and 12 weeks. Depending on the specific aim(s) of the study, the number of comparisons being made, the correlation structure of the data, and the balance between type I and type II errors, any adjustment to the p-value is to be clearly stated. – completed, line 146.

Ans. We used an unstructured correlation structure.

Line 136, the complete name of the regression test is to be stated. completed, line 144.

Ans. The change of blood pressure was performed with linear mixed-effects model (LMM) since the study measured blood pressure (BP) multiple times on each participant before and after intervention.

The accepted level of statistical significance and whether 1 or 2 tailed p value is to be stated. -completed, lines 140-141 and 151-152.

Ans. The continuous variables were analyzed using the student’s t-test with 2-tailed test.

Statistical significance was set at P-value <0.05.

Intent to treat or per protocol analysis; missing data (if any) and method of handling missing data is to stated. -completed, line 143-144.

Ans. We used an intention to treat analysis. Participants with incomplete data were excluded in the analysis.

Table 1, Table 2 and Table 3, all statistical tests are to be denoted in the table footnote. For variables ‘other underlying diseases’ and ’24-hour urine’, the figures are to be placed in the same row with their respective category.

Ans. We added P value and the figures. All statistical tests are to be denoted in the table footnote in Table 1,2 and 3 as suggested

Table 2, effect size indices and 95% CI could be presented. The p-value in the change column is to be removed and placed in a separate column of each respective group. Presentation of p-value minus the symbol = e.g. P 0.004 is to be avoided. Likewise, with Table 3. -completed, in Table 1,2 and 3.

Ensure that all the information reported in the CONSORT checklist is presented or clearly presented in the manuscript. -Completed as suggested in the manuscript.

Reviewer #2: Dear authors,
Congratulations for conducting and reporting the study. This study determines the effectiveness of a combined intervention on reducing salt intake and blood pressure relative to health education. The manuscript is well-written in standard English with sufficient data presentation, analysis, and discussion. The manuscript fulfills the stated aims and meets the standard for publication.

Strengths of the study
Authors have clearly established the research gaps and objectives. The chosen study design (cluster-randomised trial) is suitable to meet the study objectives. Data has been adequately presented and interpretated. Discussion is balanced and conclusion is based on the results and analysis.

Weaknesses of the study
Method:
Line 85: definition of hypertension is misleading. Current statement is not correct and citation is missing. -completed, line 85 of the tracked changes version.

Ans. The participants included in this study were aged between 18-70 years and had a diagnosis of hypertension (systolic blood pressure ≥130 or diastolic blood pressure ≥ 80 mmHg)[11].

Line 91: Please provide details when clusters were randomised (e.g before or after the participants were recruited) and methods for randomisation of participants. -completed, line96-102.
Ans. Each health center was responsible for 10-12 villages. We used a random number generator, to assign a random number (1, 2) to each village (cluster). The villages assigned the number 1 were the intervention group, and the villages assigned the number 2 were the control group. A total of 6 clusters were randomly allocated to the intervention group and another 6 clusters to the control group. A total of 20 patients meeting the eligible criteria were randomly recruited, using the random number generator, in each cluster. Informed consent was obtained from all participants. (Fig 1)

Line 87 and 88: The expression "During the conduction of the trial, participants were not allowed to adjust their antihypertensive or diuretic medication" is misleading. Is it ethical not to allow participants to alter the drug? -completed, lines 87-90.

Ans. If participants had systolic blood pressure exceeding 180 mmHg or presented with hypertensive emergencies, they were excluded from the study.

Line 105: Authors did not register the "knowledge, attitude, and behavior related to dietary consumption" related outcome in the trial registry. This can be considered as exploratory outcomes only.

Ans. We agree and added in the discussion lines 242-244.

Line 109 to 116: Please provide the details of intervention (e.g duration and sessions of health education), so that the intervention could be replicated. -completed, lines 115-116.

Ans. First, intensive dietary education, participants received education from nurses and dietitians about high sodium intake, sources of sodium, and nutritional ingredients for 2 hours on day 0 and for 1 hour at 4 and 8 weeks.

Results:
Table 1: *is missing in the table. What indicates * for.

Ans. We added * and P value in the table and all statistical tests are to be denoted in the table footnote in Table 1,2 and 3 as suggested

Table 2: Please report intra-cluster correlation coefficients for the reported outcomes. These are important for future studies to estimate power and meta-analyse. -completed, lines 189-190.

Ans. The intra-cluster correlation coefficients were 0.754 and 0.826 for systolic blood pressure and diastolic blood pressure respectively.

Discussion:

Line 217: "Furthermore, we observed the trend in reduction of blood pressure and 24-hour urinary sodium excretion in the control group which may be due to patient education or unknown intervention during follow-up clinic visit". It is not the limitation of the study. Instead, This is the indication that standard health education is properly implemented. Therefore, this could be discussed separately.

Ans. We agree and edited in the discussion as suggested on lines 212-214

Line 219: The generalisability is limited as the study was conducted in selected villages. -completed, line 248.

There are additional limitations. One limitation is not considering important lifestyle changes (e.g exercise). Authors did not report baseline data on history of current anti-hypertensive medication, which either could be adjusted in the analysis or acknowledged in the limitation. -completed, lines 244-246.

It would be easier to follow the structure when strength and limitations are in the last sentence of discussion section.
Ans: We agree and edited in the discussion as suggested on lines 240-248.

Limitations:

Language:
There are a few errors in English language: e.g
Line 198: Long term is Long-term -completed, line 215.

**Journal Requirements:**

When submitting your revision, we need you to address these additional requirements.

1. Please ensure that your manuscript meets PLOS ONE's style requirements, including those for file naming. The PLOS ONE style templates can be found at 
https://journals.plos.org/plosone/s/file?id=wjVg/PLOSOne_formatting_sample_main_body.pdf and 
https://journals.plos.org/plosone/s/file?id=ba62/PLOSOne_formatting_sample_title_authors_affiliations.pdf

We have checked and ensured our manuscript meets PLOS ONE’s requirements.

2. We note that you have selected “Clinical Trial” as your article type. PLOS ONE requires that all clinical trials are registered in an appropriate registry (the WHO list of approved registries is at      https://www.who.int/clinical-trials-registry-platform/network/primary-registries" https://www.who.int/clinical-trials-registry-platform/network/primary-registries and more information on trial registration is at http://www.icmje.org/about-icmje/faqs/clinical-trials-registration/).
 
Please state the name of the registry and the registration number (e.g. ISRCTN or ClinicalTrials.gov) in the submission data and on the title page of your manuscript. completed, line 15-16.
a) Please provide the complete date range for participant recruitment and follow-up in the methods section of your manuscript. completed, line 93.

b) If you have not yet registered your trial in an appropriate registry, we now require you to do so and will need confirmation of the trial registry number before we can pass your paper to the next stage of review. Please include in the Methods section of your paper your reasons for not registering this study before enrolment of participants started. Please confirm that all related trials are registered by stating: “The authors confirm that all ongoing and related trials for this drug/intervention are registered”.
 
Please see http://journals.plos.org/plosone/s/submission-guidelines#loc-clinical-trials for our policies on clinical trials.

3. Thank you for stating the following financial disclosure: 
"This study was financially supported by the Thai Health Promotion Foundation No. 64-00255-0002 and World Health Organization (WHO) office, Thailand, registration 2021/1159185-0 "

Please state what role the funders took in the study.  If the funders had no role, please state: "The funders had no role in study design, data collection and analysis, decision to publish, or preparation of the manuscript." 
If this statement is not correct you must amend it as needed. 
Please include this amended Role of Funder statement in your cover letter; we will change the online submission form on your behalf. -Completed in covering letter.

4. We note that the original protocol that you have uploaded as a Supporting Information file contains an institutional logo. As this logo is likely copyrighted, we ask that you please remove it from this file and upload an updated version upon resubmission. -Completed and new file attached
