# Supplementary material for: Community-based intervention for monitoring of salt intake in hypertensive patients: A cluster randomized controlled trial
Source: PLoS One. 2024 Nov 22;19(11):e0311908. doi: 10.1371/journal.pone.0311908 (PMC11584128; doi:10.1371/journal.pone.0311908)
Supplement: Attachment — Submitted filename: Response to reviewers.docx [file pone.0311908.s008.docx]

Reviewer #2: I only have a few minor corrections. Please make the writing of p value consistent, either p or P. All tables contain the redundant letter P, column head with P value is enough. To improve reading, all the table titles should be just before each table. These corrections can be made even during production phase.

Ans. We used P value consistently in the manuscript. We deleted P value in all the tables except column head and put the titles before each table as suggested.

Reviewer #3: The authors have adequately addressed most of the comments raised in a previous round of review. This manuscript needs further improvement.
1. The positioning of the modifications made in the author's response does not match the actual situation, resulting in reading difficulties.

Ans. We revised the manuscript which the positioning of modification in the author’s response to match the actual situation as suggested.

2. It would be preferable to use a three-line table format in the assay

Ans. We changed all tables to a three-line table format as suggested.

3. The patients recruited exhibited a significant age range, with the youngest being only 18 years old. But the exclusion criteria do not include secondary hypertension, such as primary aldosteronism. Salt intake may play a more significant role in this condition.

Ans. We recruited the patients with no identifiable causes of hypertension and randomized them so that the underlying diseases were comparable. Furthermore, most of the patients were middle-aged and elderly patients, with a mean age of 60 ± 7.5 (range 26-70) years old in intervention group and 60.2 ± 7.5 (range 36-70) in control group, which is an uncommon age group for primary aldosteronism.

4. The author provided detailed information about the randomization method and process. Line 207,“240 participants were enrolled from 6 clusters/group”, 12 clusters might be an accurate representation according to my understanding.

Ans. Participants were recruited from twelve clusters (villages) in six healthcare centers. (line 122) A total of 6 clusters were randomly allocated to the intervention group and another 6 clusters to the control group. (Line 129-131)

5.In the discussion section, the author noted that there was no adjustment of medication in patients. It is puzzling that the blood pressure in the control group still showed a significant decline.

Ans. We observed the trend in reduction of blood pressure and 24-hour urinary sodium excretion in the control group during follow-up clinic visit, indicating that standard health education was properly implemented in our study and may contribute to improvement in blood pressure control. (Line 297-300)

6.As mentioned before, the presentation of p-value should not minus the symbol = e.g. P 0.004.

Ans. We made change of P value as suggested.
