# Supplementary material for: Community-based intervention for monitoring of salt intake in hypertensive patients: A cluster randomized controlled trial
Source: PLoS One. 2024 Nov 22;19(11):e0311908. doi: 10.1371/journal.pone.0311908 (PMC11584128; doi:10.1371/journal.pone.0311908)
Supplement: Attachment — Submitted filename: Response to reviewers.docx [file pone.0311908.s009.docx]

We response to reviewers’ comments below.

Reviewer #4: In this manuscript, the authors explored the effects of salt intake monitoring in urine sodium excretion and blood pressure. However, there are some concerns need to be addressed.

1. Since the baseline body weight and BMI were statistically higher in interventional group, the changes of these characteristics after follow-up should be provided and whether the changes were correlated with improver BP should be explored.

Answer. Since the baseline body weight and BMI were statistically higher in the interventional group, we studied changes in these characteristics after 12 weeks of follow-up. There was no significant difference (P=0.09) in the change in BMI from baseline between the two groups (BMI decreased by 0.03 and 0.23 kg/m^2^ in the intervention and control groups, respectively). Therefore, the noticeable changes in blood pressure were not attributable to weight reduction. (Lines 190-194 in revised manuscript with track changes)

2. The details of medicine should be provided, especially SGLT2i.

Answer. We did not consider lifestyle changes (e.g. exercise) and baseline anti-hypertensive medications. This is due to the fact that in the rural primary healthcare setting where this study was done, there is a limited list of first-line medications for high blood pressure (thiazide diuretics, calcium channel blockers and angiotensin-converting enzyme inhibitors).  Sodium-glucose cotransporters-2 inhibitors are not available in this setting. In addition, there were no reports of any symptoms related to hypertensive complications, nor any adjustment of medications in our patients (Discussion on limitation of the study, Lines 249-254.)

3. The results should be presented with brief titles.

Answer. We put brief titles in the results as suggested.
